# A Comparative Study Using Reversed-Phase and Hydrophilic Interaction Liquid Chromatography to Investigate the In Vitro and In Vivo Metabolism of Five Selenium-Containing Cathinone Derivatives

**DOI:** 10.3390/metabo15080497

**Published:** 2025-07-23

**Authors:** Lea Wagmann, Jana H. Schmitt, Tanja M. Gampfer, Simon D. Brandt, Kenneth Scott, Pierce V. Kavanagh, Markus R. Meyer

**Affiliations:** 1 Department of Experimental and Clinical Toxicology and Pharmacology, Institute of Experimental and Clinical Pharmacology and Toxicology, Center for Molecular Signaling (PZMS), Saarland University, 66421 Homburg, Germany; 2 The Alexander Shulgin Research Institute, 1483 Shulgin Road, Lafayette, CA 94549, USA; 3 Department of Pharmacology and Therapeutics, School of Medicine, Trinity Centre for Health Sciences, St. James Hospital, D08 W9RT Dublin 8, Ireland

**Keywords:** cathinones, selenophene, LC-HRMS/MS, toxicokinetics, isozyme mapping

## Abstract

**Background/Objectives**: The emergence of cathinone-based psychostimulants necessitates ongoing research and analysis of the characteristics and properties of novel derivatives. The metabolic fate of five novel cathinone-derived substances (ASProp, MASProp, MASPent, PySProp, and PySPent) containing a selenophene moiety was investigated in vitro and in vivo. **Methods**: All compounds were incubated individually with pooled human liver S9 fraction. A monooxygenase activity screening investigating the metabolic contribution of eleven recombinant phase I isoenzymes was conducted. Rat urine after oral administration was prepared by urine precipitation. Liquid chromatography–high-resolution tandem mass spectrometry was used for the analysis of all samples. Reversed-phase liquid chromatography (RPLC) and zwitterionic hydrophilic interaction liquid chromatography (HILIC) were used to evaluate and compare the metabolites’ chromatographic resolution. **Results**: Phase I reactions of ASProp, MASProp, MASPent, PySProp, and PySPent included *N*-dealkylation, hydroxylation, reduction, and combinations thereof. The monooxygenase activity screening revealed the contribution of various isozymes. Phase II reactions detected in vivo included *N*-acetylation and glucuronidation. Both chromatographic columns complemented each other. **Conclusions**: All substances revealed metabolic reactions comparable to those observed for other synthetic cathinones. Contributions from isozymes to their metabolism minimized the risk of drug–drug interactions. The identified metabolites should be considered as targets in human biosamples, especially in urine screening procedures. RPLC and HILIC can both be recommended for this purpose.

## 1. Introduction

New psychoactive substances (NPSs) have been an integral part of the market for drugs of abuse for more than a decade now. According to the latest World Drug Report, the United Nations Office on Drugs and Crime identified 44 NPSs for the first time and monitored a total of 566 different NPSs in 2022 [1]. Thirty-five percent of these NPSs were declared to be stimulants, a drug class that is largely composed of amphetamine-type and cathinone-derived compounds. The latter are synthetic analogues of cathinone, the major active ingredient of the kath plant *Catha edulis* [2]. Detections and seizures of synthetic cathinones have raised significant concerns, as their misuse is linked to potent psychoactive effects and unpredictable health risks. Ingestion may lead to a range of serious effects, including aggression, agitation, paranoia, delusions, seizures, hyperthermia, rhabdomyolysis, renal and hepatic failure, and, in some cases, death [3]. When compared to amphetamines, synthetic cathinones share a β-keto moiety in their structure and act as psychomotor stimulants that typically increase extracellular monoamine concentrations in the brain involving plasma membrane transporters for dopamine, norepinephrine, and serotonin [2]. Whether they increase neurotransmitter concentrations by release or reuptake is largely dependent on their chemical structure [2]. This fact, as well as the diversity of chemical structures of synthetic cathinones available in the market for drugs of abuse, complicates the prediction of the toxicological effects of emerging NPS.

A comprehensive understanding of the toxicodynamic and toxicokinetic properties of NPSs is essential for evaluating their characteristics. In addition, this knowledge will also support the assessment of risks and the formulation of appropriate analytical screening procedures. In order to determine whether NPSs are involved in fatal or non-fatal intoxications, it is essential that screenings are available within clinical and forensic toxicology to detect the parent compound and/or its metabolites in human biosamples. Therefore, understanding the metabolic pathways of NPSs is a crucial prerequisite, particularly for the development of urine screening methodologies [4].

**Figure 1:**
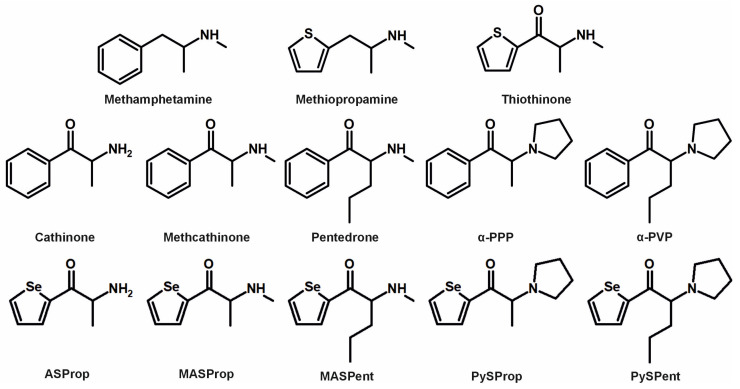
Chemical structures of methamphetamine, methiopropamine, thiothinone, cathinone, methcathinone, pentedrone, α-PPP, α-PVP, and the five selenium-containing cathinone derivatives.

Although cathinone-based psychostimulants are well-documented substances of abuse, an overview of the NPS phenomenon reveals that a variety of newly emergent stimulants exhibit structural modifications that adhere to established principles in drug design. One such example involves a bioisosteric replacement, as found, for example, in *N*-methyl-1-(thiophen-2-yl)propan-2-amine (methiopropamine, 2-MPA, MPA, Figure 1). In this instance, the phenyl ring present in methamphetamine is substituted with a thiophene moiety, while still exhibiting stimulant activity [5]. Examples such as this and other cathinones suggest that the motivation for introducing NPSs into the market was influenced by information disseminated in the scientific literature [5,6]. Correspondingly, the β-keto counterpart of methiopropamine (bk-MPA; 2-(methylamino)-1-(thiophen-2-yl)propan-1-one; thiothinone) could be seen as a methcathinone derivative, and its detection was reported in seized material [7], which demonstrates the necessity of monitoring such developments. Understanding the pharmacological and toxicokinetic features of novel drugs that could emerge in the NPS market also led to the concept of investigating “prophetic” drugs to enhance strategic advantages in the cat-and-mouse game within the drugs field [8].

Similarly, the present study concentrated on exploring novel derivatives by substituting thiophene (or the phenyl ring) with selenophene. The chemical reactivity of selenophene has been described as similar to that of other five-membered heterocycles, such as thiophene [9]. Substances containing selenium have attracted considerable attention in recent years [10,11,12] and compounds containing a selenophene ring system have been reported to exhibit a wide range of biological activities, including antioxidative, antinociceptive, and anti-inflammatory effects [9]. However, the extent to which selenophene-derived cathinone counterparts exhibit psychostimulant properties remains uncertain. The chosen selenophene cathinone derivatives used for this initial study are summarized in Figure 1 and included 2-amino-1-(selenophene-2-yl)propan-1-one (ASProp), 1-(selenophene-2-yl)-2-(methylamino)propan-1-one (MASProp), 1-(selenophene-2-yl)-2-(methylamino)pentan-1-one (MASPent), 1-(selenophene-2-yl)-2-(pyrrolidin-1-yl)propan-1-one (PySProp), and 1-(selenophene-2-yl)-2-(pyrrolidin-1-yl)pentan-1-one (PySPent). The compounds were chosen to reflect analogs of known drugs of abuse that feature a phenyl ring in place of the selenophene moiety (all structures depicted in Figure 1). These include cathinone, methcathinone, pentedrone, alpha-pyrrolidinopropiophenone (also known as α-PPP), and alpha-pyrrolidinopentiophenone (also commonly referred to as alpha-pyrrolidinovalerophenone or α-PVP). As mentioned above, for MASProp and PySPent, there are analogous drugs of abuse available on the market that incorporate a thiophene ring instead of the selenophene moiety. In addition to thiothinone, alpha-pyrrolidinopentiothiophenone (α-PVT) is a known NPS. The selenophene-containing compounds may be regarded as non-classical bioisosteres of the drugs of abuse containing a phenyl or a thiophene ring.

To the best of our knowledge, neither these compounds nor other selenophene-containing NPSs were detected in the market for drugs of abuse to date, and nothing is known about the biological properties of the selenophene-containing cathinone derivatives in comparison to their analogs. Preliminary data indicate that MASPent and PySPent, in particular, are potent dopamine reuptake inhibitors, but further studies are needed before publication. It was hypothesized that similarities might exist in their metabolic transformation when compared to compounds containing thiophene or phenyl rings. Thus, this study aimed to elucidate the metabolic fate of the five cathinone-derived compounds ASProp, MASProp, MASPent, PySProp, and PySPent in incubations with pooled human liver S9 (pHLS9) fraction and rat urine after oral administration. A monooxygenase activity screening should be performed to identify the isozymes involved in the initial phase I metabolic steps. Considering the heterogenous chemical properties of the compounds, all samples should be analyzed by reversed-phase liquid chromatography (RPLC) and zwitterionic hydrophilic interaction liquid chromatography (HILIC), both coupled to high-resolution tandem mass spectrometry (HRMS/MS), for evaluation and comparison of the metabolites’ chromatographic resolution.

## 2. Materials and Methods

### 2.1. Chemicals and Enzymes

Acetyl coenzyme A, dithiothreitol, dipotassium hydrogen phosphate, isocitrate, isocitrate dehydrogenase, magnesium chloride (MgCl_2_), 3′-phosphoadenosine-5′-phosphosulfate (PAPS), potassium dihydrogen phosphate, reduced glutathione, *S*-(5′-adenosyl)-l-methionine (SAM), superoxide dismutase, and Tris hydrochloride were obtained from Sigma-Aldrich (Taufkirchen, Germany), nicotinamide adenine dinucleotide phosphate (NADP^+^) from Biomol (Hamburg, Germany), and trimipramin-d3 from LGC (Wesel, Germany). Water was purified with a filtration unit, purifying water to a resistance of 18.2 Ω × cm, from Millipore (Merck, Darmstadt, Germany). All other chemicals and reagents were obtained from VWR (Darmstadt, Germany), including acetonitrile (LC-MS grade), ammonium acetate (analytical grade), ammonium formate (analytical grade), formic acid (LC-MS grade), and methanol (LC-MS grade).

The baculovirus-infected insect cell microsomes (Supersomes) containing the human complementary cDNA-expressed cytochrome P450 (CYP) isoenzymes CYP1A2, CYP2B6, CYP2C8, CYP2C19, CYP2D6, CYP3A4, CYP3A5 (1 nmol/mL, each), CYP2A6, CYP2C9, or CYP2E1 (2 nmol/mL, each), or the flavin-containing monooxygenase 3 (FMO3, 5 mg/mL), and pooled human liver microsomes (pHLM, 20 mg microsomal protein/mL, 360 pmol total CYP/mg protein, comprising 35 donors), pHLS9 (20 mg microsomal protein/mL, comprising 8 donors), uridine 5′-diphospho-glucuronosyltransferase (UGT) reaction mixture solution A (25 mM uridine 5′-diphospho-glucuronic acid), and UGT reaction mixture solution B (250 mM Tris-HCl, 40 mM MgCl_2_, and 125 µg/mL alamethicin) were obtained from Corning (Amsterdam, The Netherlands). All enzyme preparations were thawed at 37 °C after delivery, aliquoted, snap-frozen in liquid nitrogen, and stored at −80 °C until use.

### 2.2. Synthesis Procedures

ASProp, MASProp, MASPent, PySProp, and PySPent were synthesized based on standard methods reported previously [6,13]. Selenophene served as the starting material and underwent Friedel–Crafts acylation followed by α-bromination of the ketone intermediate. Subsequent amination and conversion to the hydrochloride salt forms completed the reaction sequence. The identity of all target molecules was confirmed by routine characterization including various mass spectrometric and spectroscopic methods including gas chromatography and nuclear magnetic resonance spectroscopy, and purity was determined to be ≥95%. Stock solutions of ASProp, MASProp, MASPent, PySProp, and PySPent were prepared in methanol (1 mg/mL) and stored at −20 °C.

### 2.3. Nuclear Magnetic Resonance Spectroscopy

Prepared pure substances of ASProp, MASProp, MASPent, PySProp, and PySPent were individually dissolved in DMSO-d6 and run on a Bruker (Coventry, UK) Avance III 400 NMR (^1^H at 400.13 MHz; ^13^C/DEPT-135 at 100.6 MHz) at a temperature of 298.2 K. Full spectra have been added as Figure S1 to the Supplementary Material.

### 2.4. pHLS9 Incubations

Incubation conditions were in accordance with those described in a published study [14]. More details may be found in the Supporting Information.

### 2.5. Rat Urine Collection and Sample Preparation

Animal experiments and rat urine sample preparation were in accordance with the conditions described in a published study [14]. More details may be found in the Supporting Information.

### 2.6. Monooxygenase Activity Screening

The incubation procedure was in accordance with a previous study with minor modifications [14]. More details may be found in the Supporting Information.

### 2.7. LC-HRMS/MS and Data Evaluation Conditions

Analyses were performed using a Thermo Fisher scientific (TF, Dreieich, Germany) Dionex UltiMate 3000 Rapid Separation (RS) UHPLC system with a quaternary UltiMate 3000 RS pump and an UltiMate 3000 RS autosampler controlled by the TF Chromeleon software version 6.80. The pump and autosampler were coupled to a TF Q Exactive Plus Orbitrap mass spectrometer with a heated electrospray ionization II source (HESI-II). A Positive Mode Cal Mix (Supelco, Bellefonte, PA, USA) was used for calibration before analysis.

RPLC conditions were as follows: TF Accucore Phenyl-Hexyl column (100 mm × 2.1 mm inside diameter, ID, 2.6 µm particle size) at 40 °C maintained by a Dionex UltiMate 3000 RS analytical column heater; gradient elution with 2 mM aqueous ammonium formate solution containing 0.1%, *v/v*, formic acid (eluent A) and 2 mM ammonium formate solution with acetonitrile/methanol, 50:50, *v/v*, containing 0.1%, *v/v*, formic acid and 1%, *v/v*, water (eluent B). The following gradient and settings were used: 0–2 min hold 99% A, 2–10 min to 40% A, 10–11.5 min hold 1% A, and 11.5–13.5 min hold 99% A with curve 5 for all steps. The flow rate settings were as follows: 0–10 min hold 0.500 mL/min, 10–11.5 min to 0.800 mL/min, and 11.5–13.5 min hold 0.800 mL/min.

HILIC conditions were as follows: Merck SeQuant ZIC-cHILIC column (150 mm × 2.1 mm ID, 3 μm, 100 Å) at 40 °C; gradient elution with 200 mM aqueous ammonium acetate solution, adjusted to pH 5 with acetic acid (eluent C) and acetonitrile containing 0.1% (*v/v*) formic acid (eluent D). The following gradient and settings were used: 0–2 min hold 3% C, 2–10 min to 60% C, 10–11.5 min hold 60% C, and 11.5–13.5 min hold 3% C with curve 5 for all steps. The flow rate was set to 0.500 mL/min. The injection volume was 1 µL for all samples. The HESI-II conditions and settings for full scan data acquisition with subsequent dd-MS^2^ are given in Table S1 in the Supporting Information. One out of five inclusion lists containing mass-to-charge ratios *m/z* (M+H^+^) of ASProp (*m/z* 203.9922), MASProp (*m/z* 218.0079), MASPent (*m/z* 246.0392), PySProp (*m/z* 258.0392), or PySPent (*m/z* 286.0705) and the corresponding expected metabolites were used. ChemSketch 2021 2.1 (ACD/Labs, Toronto, ON, Canada) was employed for the depiction of the structures of hypothetical metabolites and for the calculation of their exact masses. TF Xcalibur Qual Browser software version 4.5 was used for data evaluation.

## 3. Results and Discussion

### 3.1. Identification of Metabolites

**Figure 2:**
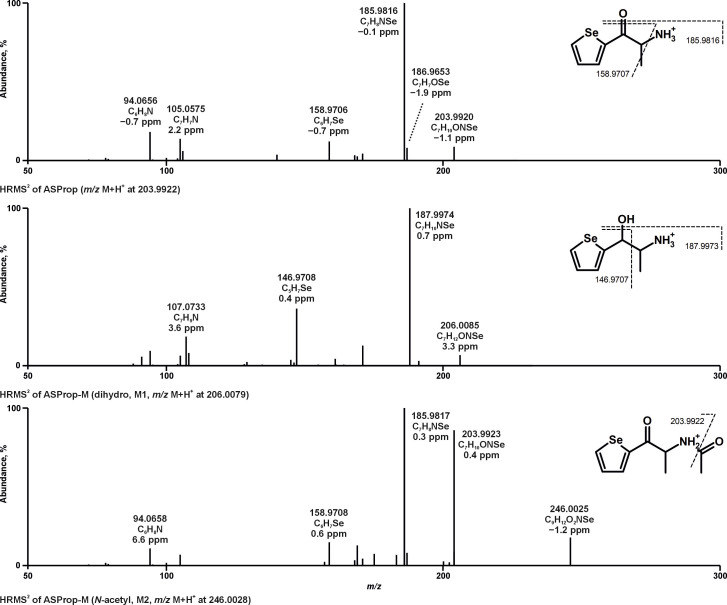
HRMS^2^ spectra of ASProp, dihydro ASProp, and *N*-acetyl ASProp detected in in vitro incubations or rat urine. Metabolite IDs correspond to Table S2 in the Supporting Information.

Metabolites of ASProp, MASProp, MASPent, PySProp, and PySPent formed after pHLS9 exposure or rat administration were tentatively identified after mass spectrometric analysis by screening MS^1^ data for exact precursor masses (PMs) of expected metabolites and subsequent evaluation of the MS^2^ spectra and a maximum deviation of 5 ppm between measured and calculated exact PMs was deemed acceptable. The fragmentation patterns in their MS^2^ spectra were interpreted and compared to those of the parent compounds and an example can be found in Figure 2. Metabolites were assigned to a unique metabolite identification number (ID) after being sorted by parent compound, increasing mass, and additionally by increasing retention time (RT) after RPLC in the case of isomers. Analytical information of the parent compounds and all phase I and II metabolites including characteristic fragment ions (FIs) with elemental compositions and relative intensities in MS^2^ is listed in Table S2 in the Supporting Information, which also contains the metabolite ID, metabolic reactions, information about whether the metabolite was detected in vitro and/or in vivo, and the RT after RPLC and HILIC. No metabolites were detected after pHLS9 incubation with 25 µM ASProp. Therefore, the incubation was repeated with 50 µM ASProp. In total, the analyses of pHLS9 incubations and rat urine resulted in detections of 38 tentative metabolites: 2 metabolites of ASProp, 5 metabolites of MASProp, 6 metabolites of MASPent, 11 metabolites of PySProp, and 14 metabolites of PySPent. The overall number of metabolites detected in pHLS9 incubations or in rat urine was comparable (29 in pHLS9, 26 in rat urine). Twelve metabolites were only detected in pHLS9 incubations, nine only in rat urine, and seventeen in both. Only 5 out of 38 metabolites identified in total were phase II metabolites, which were exclusively detected in rat urine, except for one metabolite (M34, glucuronidated hydroxy PySPent) which was also detected in pHLS9 incubations. The phase II metabolic reactions included *N*-acetylation (M2, ASProp or *N*-demethylated MASProp) and glucuronidation (M22, hydroxylated PySProp; M33 and M34, hydroxylated PySPent). Both metabolic reactions were already described as metabolic pathways of other NPS including cathinone derivatives [4,14,15,16]. Fragmentation patterns of selected compounds are discussed exemplarily in the following and the given masses in the text are the calculated exact masses.

As depicted in Figure 2, the initial fragmentation steps of the parent compound ASProp (PM at *m/z* 203.9922, C_7_H_10_ONSe) included the loss of ammonia (−17 u, NH_3_, FI at *m/z* 186.9657, C_7_H_7_OSe) or the loss of water (−18 u, H_2_O, FI at *m/z* 185.9816, C_7_H_8_NSe). The latter was described to be a characteristic fragmentation step of cathinone derivatives and resulted in the most intense FI in the MS^2^ spectrum of ASProp [17]. The additional loss of one carbon atom led to the FI at *m/z* 158.9707 (C_6_H_7_Se). The FI at *m/z* 105.0573 (C_7_H_7_N) and *m/z* 94.0651 (C_6_H_8_N) indicated the cleavage of the selenophene ring. After reduction of the carbonyl group (M1, PM at *m/z* 206.0079, C_7_H_12_ONSe), the elimination of water (−18 u, H_2_O) resulted in a shift of +2 u from FI at *m/z* 185.9816 (C_7_H_8_NSe for ASProp) to 187.9973 (C_7_H_10_NSe for M1). The same was true for the FI at *m/z* 105.0573 (C_7_H_7_N for ASProp) compared to the FI at *m/z* 107.0730 (C_7_H_9_N for M1). The initial step during the fragmentation of *N*-acetyl ASProp (M2, PM at *m/z* 246.0028, C_9_H_12_O_2_NSe) was the elimination of the acetyl moiety resulting in the FI at *m/z* 203.9922 (C_7_H_10_ONSe). The following fragmentation corresponded to that of the parent compound.

The initial fragmentation step of the parent compound MASPent (PM at *m/z* 246.0391, C_10_H_16_ONSe) was the loss of water (−18 u, H_2_O) resulting in the FI at *m/z* 228.0285 (C_10_H_14_NSe). This FI was subsequently split in two leading to the FI at *m/z* 144.9550 (C_5_H_5_Se) and the most intense FI at *m/z* 86.0964 (C_5_H_12_N). After *N*-demethylation (M7, PM at *m/z* 232.0234, C_9_H_14_ONSe), the loss of ammonia (−17 u, NH_3_) led to the FI at *m/z* 214.9969 (C_9_H_11_OSe), while the elimination of water (−18 u, H_2_O) resulted in the FI at *m/z* 214.0129 (C_9_H_12_NSe). The latter was subsequently split into the FI at *m/z* 144.9550 (C_5_H_5_Se) and the most intense FI at *m/z* 72.0807 (C_4_H_10_N). Three peaks with the PM at *m/z* 262.0340 (M10-12, C_10_H_16_O_2_NSe) were detected, indicating a hydroxylation. In the case of M10 and M11, the hydroxy group was found to be located at the selenophene ring due to the shift of +16 u from FI at *m/z* 144.9550 (C_5_H_5_Se for MASPent) to 160.9500 (C_5_H_5_OSe for M10 and M11). Due to the chromatographic separation of M10 and M11, different positions of the hydroxy groups are likely, but the identification of the exact positions was not possible based on fragmentation patterns. In the case of M12, the selenophene ring was not hydroxylated due to the presence of the FI at *m/z* 144.9550 (C_5_H_5_Se). However, the FI at *m/z* 86.0964 (C_5_H_12_N for MASPent) was shifted to 102.0913 (C_5_H_12_ON for M12). The RT of M12 after RPLC analysis was higher than the RT of MASPent. This fact may indicate the formation of a hydroxylamine. The *N*-oxygenation of the nitrogen atom was already described for other cathinone derivatives, as well as for methamphetamine [4,18].

The MS^2^ spectrum of the parent compound PySPent (PM at *m/z* 286.0705, C_13_H_20_ONSe) did not exhibit a loss of water, which is in line with previous reports on the fragmentation of pyrrolidinophenones [4,17,19]. After elimination of the pyrrolidine ring (−71 u, C_4_H_9_N), the FI at *m/z* 214.9970 (C_9_H_11_OSe) was formed. The FI at *m/z* 158.9343 (C_5_H_3_OSe) represented the selenophenemethanone moiety formed after alpha cleavage, and the FI at *m/z* 144.9550 (C_5_H_5_Se) represented the selenophene ring plus methylene group, but without oxygen. The FI at *m/z* 126.1277 (C_8_H_16_N) exhibited the highest intensity and represented the pyrrolidine ring connected to a butylene chain formed after alpha cleavage. The pyrrolidine ring was detected as the FI at *m/z* 72.0808 (C_4_H_10_N) for the protonated ring and the FI at *m/z* 70.0651 (C_4_H_8_N) for the protonated dehydro pyrrolidine ring in accordance with other pyrrolidinophenones [19]. Two peaks with the PM at *m/z* 300.0497 (M24 and M25, C_13_H_18_O_2_NSe) were detected, indicating a hydroxylation and subsequent oxidation. Both MS^2^ contained the same fragments but with different intensities. Alpha cleavage led to the FI at *m/z* 144.9550 (C_5_H_5_Se) representing the selenophene ring plus methylene group and the FI at *m/z* 140.1070 (C_8_H_14_ON) representing the pyrrolidine ring with the oxo group connected to the butylene chain. The FI at *m/z* 86.0600 (C_4_H_8_ON) represented the pyrrolidine ring with the oxo group and was shifted by +14 u in comparison to the FI at *m/z* 72.0808 (C_4_H_10_N) in the MS^2^ spectrum of PySPent. Although the identification of the exact position of the oxo group was not possible based on fragmentation patterns, the differences in the RT after RPLC analysis may be used for further interpretation. While M24 showed almost the same RT as PySPent, M25 eluted more than three minutes later. Due to this fact and a comparison with the literature, the oxo group of M25 is expected to be positioned next to the nitrogen atom, resulting in a lactam [4,19]. Four metabolites with the PM at *m/z* 302.0654 (M26-29, C_13_H_20_O_2_NSe) were detected. In the case of M27, the hydroxy group was found to be located at the selenophene ring due to the shift of +16 u from the FI at *m/z* 144.9550 (C_5_H_5_Se for PySPent) to 160.9500 (C_5_H_5_OSe for M27). As the hydroxy groups of M26 and M28 were found to be located at the pyrrolidine ring, with the FI at *m/z* 70.0651 (C_4_H_8_N for PySPent) being shifted by +16 u to 86.0600 (C_4_H_8_ON for M26 and M28), both metabolites are expected to be the precursors of M24 and M25. The FI at *m/z* 86.0600 (C_4_H_8_ON) was also present in the MS^2^ spectrum of M29 and the fragmentation pattern together with the RT after RPLC analysis, which was higher than the RT of PySPent, led to the conclusion that this metabolite was an *N*-oxide. Concerning phase II metabolites of PySPent, two *O*-glucuronides (M33 and M34) could be detected (PM at *m/z* 478.0975, C_19_H_28_O_8_NSe) and their fragmentation patterns were in accordance with the corresponding phase I metabolites (M27 and M28, respectively) after elimination of glucuronic acid (−176 u, C_6_H_8_O_6_). The interpretation of the MS^2^ spectra of all other tentatively identified metabolites was performed accordingly. MASProp (PM at *m/z* 218.0078, C_8_H_12_ONSe), PySProp (PM at *m/z* 258.0392, C_11_H_16_ONSe), and their metabolites showed similar fragmentation patterns.

### 3.2. Metabolic Pathways and Isozyme Mapping

**Figure 3:**
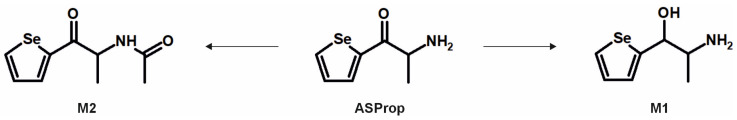
Metabolic pathways of ASProp detected in in vitro incubations or rat urine. Metabolite IDs correspond to Table S2 in the Supporting Information. Metabolic reactions are indicated by arrows.

**Figure 4:**
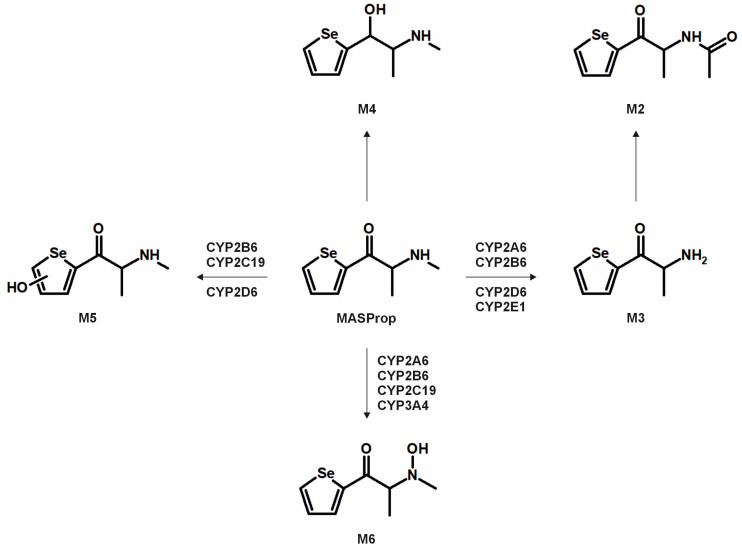
Metabolic pathways of MASProp detected in in vitro incubations or rat urine. Metabolite IDs correspond to Table S2 in the Supporting Information. Metabolic reactions are indicated by arrows. Human monooxygenases identified to be involved in the formation of the given single-step phase I metabolites during the monooxygenase activity screening are indicated.

**Figure 5:**
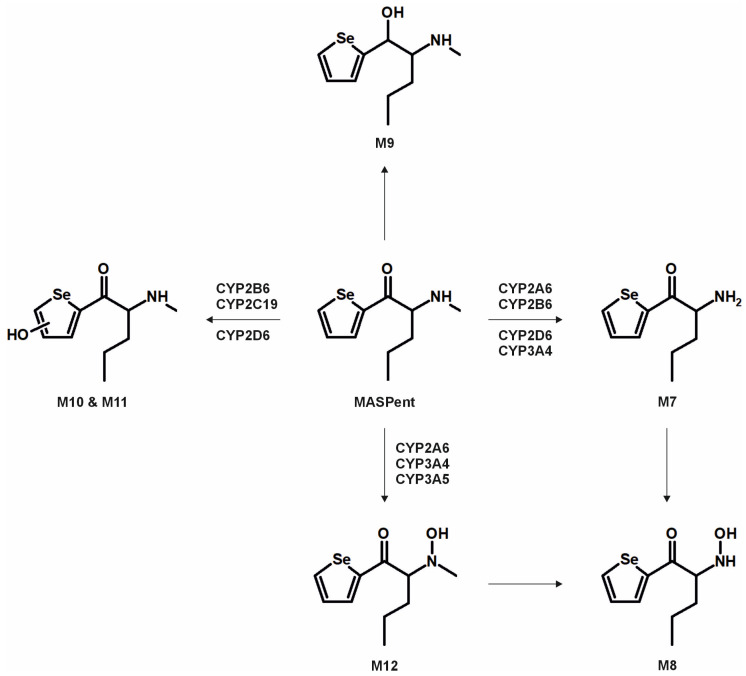
Metabolic pathways of MASPent detected in in vitro incubations or rat urine. Metabolite IDs correspond to Table S2 in the Supporting Information. Metabolic reactions are indicated by arrows. Human monooxygenases identified to be involved in the formation of the given single-step phase I metabolites during the monooxygenase activity screening are indicated.

**Figure 6:**
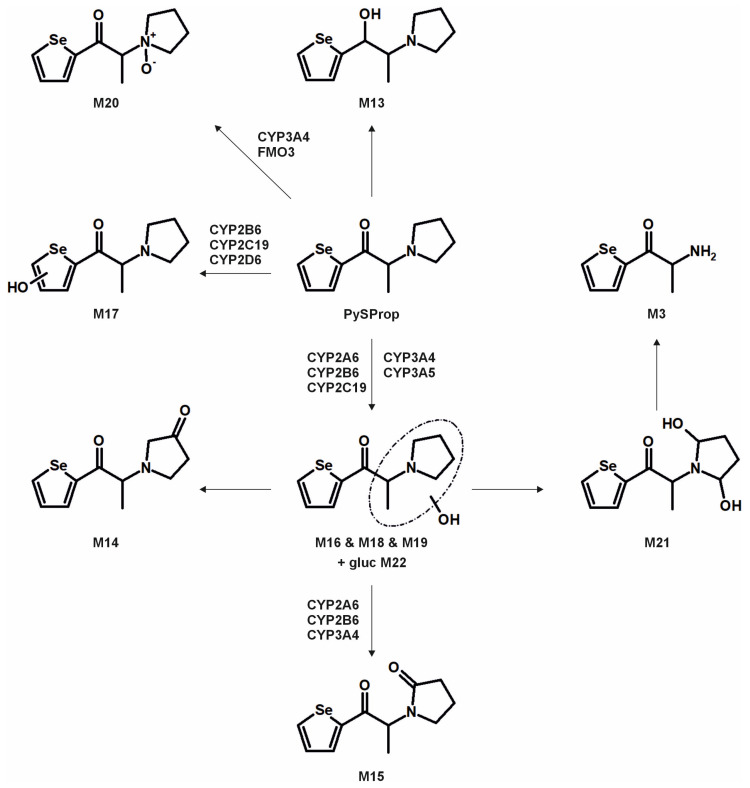
Metabolic pathways of PySProp detected in in vitro incubations or rat urine. Metabolite IDs correspond to Table S2 in the Supporting Information. Metabolic reactions are indicated by arrows. Human monooxygenases identified to be involved in the formation of the given single-step phase I metabolites during the monooxygenase activity screening are indicated.

**Figure 7:**
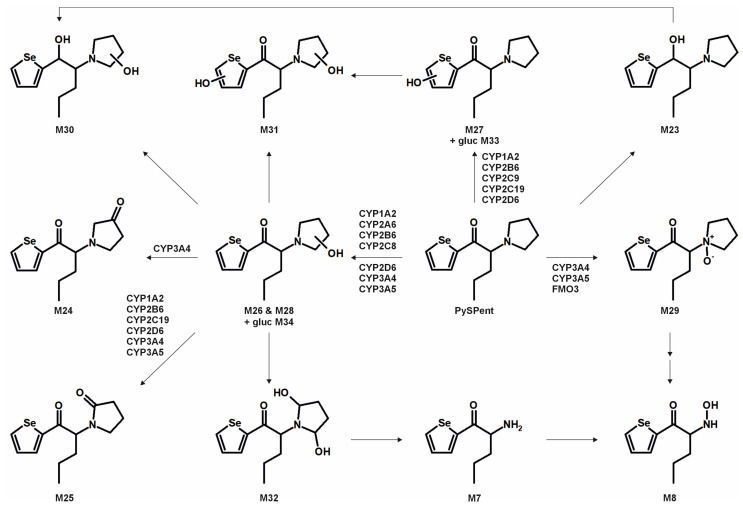
Metabolic pathways of PySPent detected in in vitro incubations or rat urine (gluc: glucuronic acid). Metabolite IDs correspond to Table S2 in the Supporting Information. Metabolic reactions are indicated by arrows. Human monooxygenases identified to be involved in the formation of the given single-step phase I metabolites during the monooxygenase activity screening are indicated.

The metabolic pathways of ASProp, MASProp, MASPent, PySProp, and PySPent can be found in Figure 3, Figure 4, Figure 5, Figure 6 and Figure 7. The results of the current study confirmed the hypothesis that similarities exist in their metabolic transformation when compared to compounds containing thiophene or phenyl rings, and the depicted metabolic reactions were already described as metabolic pathways of other phenethylamine or cathinone derivatives [4,15,16,19,20,21].

The monooxygenase activity screening was used to investigate the role of eleven phase I isoenzymes (ten CYP isoforms and FMO3) in the phase I metabolism of the five compounds. If only single isozymes are involved in the metabolism of a compound, this might indicate possible drug–drug or drug–food interactions by enzyme inhibition or interindividual differences in the enzyme activities. This may result in reduced elimination from the body, leading to enhanced toxicity in certain individuals. Incubations with pHLM were used as a positive control. No metabolites were detected after 30 min of incubation of ASProp. Therefore, the ASProp incubation was repeated and prolonged to 60 min. Isozymes successfully identified in the monooxygenase activity screening as being involved in single-step phase I reactions are summarized in Figure 4, Figure 5, Figure 6 and Figure 7 and Table S3 in the Supporting Information. Phase I metabolic reactions included the reduction of the carbonyl group and hydroxylation, partially followed by further oxidation, *N*-oxygenation, and *N*-dealkylation. The isozyme mapping revealed that the reduction of the carbonyl group was most likely catalyzed by other metabolic enzymes as none of the tested monooxygenases was involved in the formation of dihydro ASProp (M1), dihydro MASProp (M4), dihydro MASPent (M9), dihydro PySProp (M13), and dihydro PySPent (M23). However, this was consistent with results reported from biotransformations involving other cathinone derivatives [4]. Hydroxylations were mainly catalyzed by CYP2B6, CYP2C19, CYP2D6, and CYP3A4, which were also involved in the subsequent formation of oxo metabolites. Only M14 (oxo PySProp isomer 1), which was tentatively identified during rat urine analysis, could not be detected in pHLM or any single enzyme incubation. The *N*-oxygenation of MASProp and MASPent was mainly catalyzed by CYP2A6 and CYP3A4, and that of PySProp and PySPent by CYP3A4 and FMO3. *N*-Dealkylations were mainly catalyzed by CYP2A6, CYP2B6, CYP2D6, and CYP3A4. In summary, various isozymes were involved in the formation of the phase I metabolites of MASProp, MASPent, PySProp, and PySPent, reducing the risk of hampered elimination and increased toxicity due to interactions or interindividual enzyme expression differences.

### 3.3. Comparison of Analyte Separation by RPLC or HILIC

RPLC and HILIC are both suitable techniques for the chromatographic separation of compounds present in biological matrices prior to mass spectrometric analysis. While HILIC has gained increasing attention, particularly for the quantitative determination of polar drugs and their metabolites [22,23], RPLC is still considered the gold standard for toxicological screening in clinical and forensic toxicology. To date, the literature describing the combined use of both RPLC and HILIC for the tentative identification of human metabolic biomarkers of drugs of abuse relevant to clinical and forensic toxicology is lacking. The aim of the present study was therefore to evaluate the chromatographic resolution of five selenium-containing cathinone derivatives and their metabolites using both chromatographic systems. By comparing their retention behavior, we sought to assess whether HILIC may serve as a useful complement or even a potential alternative to RPLC for future metabolism studies and screening procedures involving NPS.

**Figure 8:**
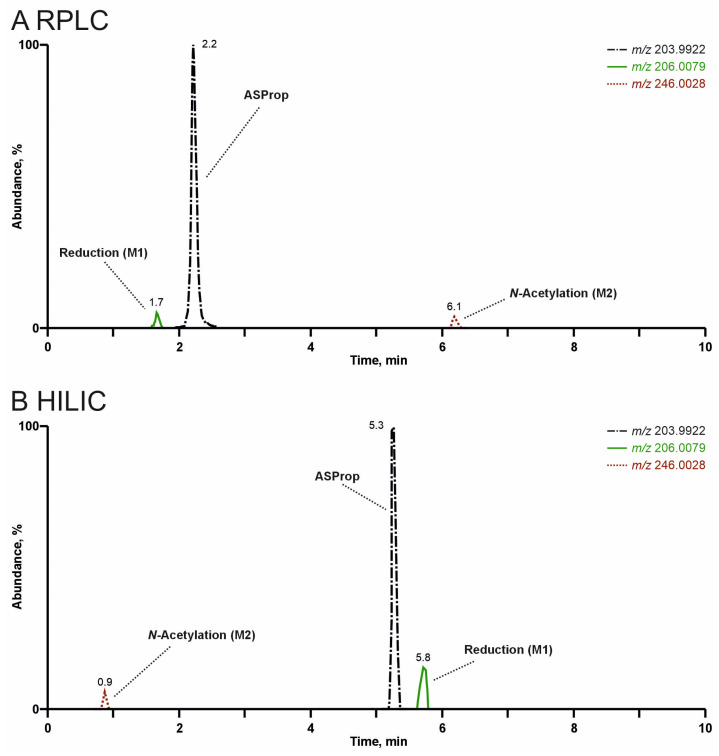
Extracted ion chromatograms of ASProp and its metabolites (M1 and M2) after high-resolution tandem mass spectrometry analysis of rat urine precipitates by reversed-phase liquid chromatography (RPLC, see (**A**)) or zwitterionic hydrophilic interaction liquid chromatography (HILIC, see (**B**)).

In general, both liquid chromatography columns complemented each other. The RT values of the parent compounds were between 2.2 min (ASProp) and 5.8 min (PySPent) using RPLC, and between 0.9 min (PySPent) and 5.3 min (ASProp) using HILIC. The chromatographic separation of ASProp and its metabolites by RPLC or HILIC is depicted in Figure 8. While dihydro ASProp (M1) eluted earlier than ASProp, *N*-acetyl ASProp (M2) eluted later than the parent compound using RPLC (see 5A). The retention order using HILIC was the reverse (see 5B).

**Figure 9:**
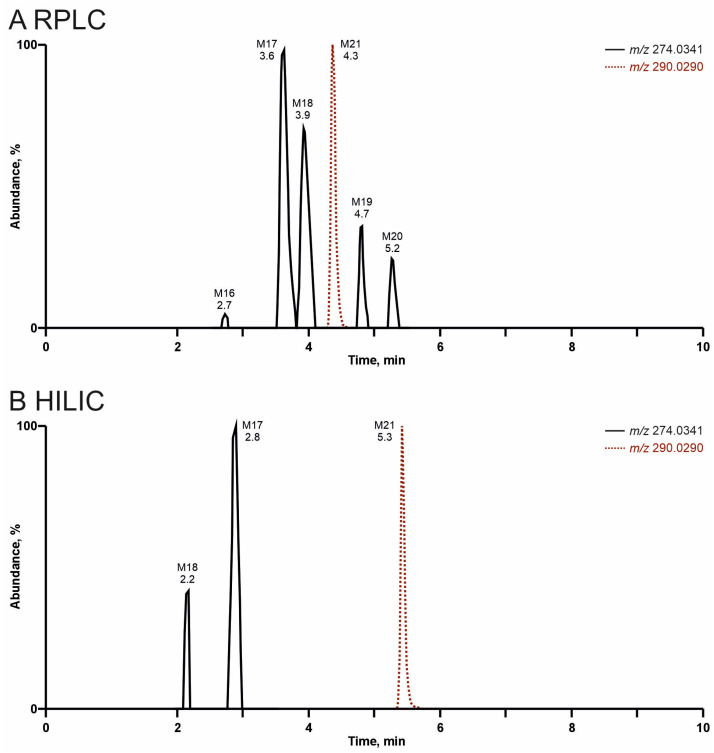
Extracted ion chromatograms of PySProp metabolites formed after mono- (M16-M20, *m/z* 274.0341) or dihydroxylation (M21, *m/z* 290.0290) after high-resolution tandem mass spectrometry analysis of pHLS9 incubations (360 min) by reversed-phase liquid chromatography (RPLC, see (**A**)) or zwitterionic hydrophilic interaction liquid chromatography (HILIC, see (**B**)).

As expected, HILIC was well suited to achieve higher RT values for small, polar analytes and to separate highly polar metabolites, while RPLC was superior in the analysis of larger, apolar molecules. However, because most analytes contained apolar and polar groups, retention on both columns was possible in general. Thus, the separation and tentative identification of most metabolites would have been successful by using either of the two mentioned chromatographic approaches. However, the tentative identification of some minor metabolites was only successful due to the better chromatographic behavior associated with higher peaks in MS^1^ and better FI intensities in MS^2^ either after RPLC or HILIC. One example is illustrated in Figure 9 for metabolites of PySProp after pHLS9 incubation being products of mono- (M16-M20) or dihydroxylation (M21). Six metabolites were detectable after RPLC separation (see Figure 9A). However, these metabolites showed varying peak intensities, most probably due to differences in concentrations and/or ionization efficiencies. After HILIC, only three metabolites could be tentatively identified. The dihydroxy metabolite M21 eluted one minute later, while M17 and M18 showed lower RT values, underlining different interaction mechanisms with the column material. The HILIC RT values of M16, M19, and M20 given in Table S2 in the Supporting Information could only be determined by analyzing rat urine precipitates and/or pHLM incubations. This is also true for several other metabolites and provides an explanation of why Table S2 in the Supporting Information contains RT on both columns for all metabolites. Only the combination of both chromatographic approaches enabled the tentative identification of 38 metabolites in total.

Nevertheless, some limitations of this study must be taken into account. The tested columns differ not only in their stationary phase, but also in length and particle size, which may influence the chromatographic resolution of the compounds. Furthermore, it is noteworthy that for nine metabolites, HILIC RT values of less than one minute were observed. These RT values (ranging from 0.8 to 0.9 min) are close to or equal to the system’s hold-up time, indicating that these compounds were not retained by the stationary phase. As a result, ion suppression and co-elution are likely to impair the ability to reliably identify these metabolites. Finally, it must be noted that the buffers were not checked for suppressive effects on the ion signals, nor was the measurement in negative ionization mode tested for analysis of metabolites, e.g., after metabolic loss of nitrogen.

### 3.4. Comparison of Metabolism Models and Screening Targets

The current study combined an in vivo animal experiment with in vitro incubations using human liver cell fractions to identify suitable urine screening targets. The intake by humans in the framework of a controlled trial would be the gold standard for identifying bioanalytical targets and developing urine screening approaches, but such trials are time consuming, expensive, and considered unethical, especially in the context of drugs of abuse due to the incalculable health risk [4]. The use of human tissue fractions represents a well-established alternative, but in vitro experiments always have limitations concerning distribution and excretion, potentially limiting the variety of metabolites and leading to the preference of simple metabolites formed after few reaction steps. While rat urine was collected over a 24 h period, the pHLS9 incubations were terminated after 1 h or 6 h. Richter et al. demonstrated that an incubation time of 6 h was sufficient for the formation of multistep reaction metabolites [4] but usually with lower signal intensities compared to in vivo approaches.

Furthermore, species differences can cause the formation of different metabolic patterns and even a minor metabolite in rats may be found to be a major metabolite in humans [24]. Nevertheless, rats are the most commonly used mammalian model for the identification of urinary biomarkers of new drugs of abuse. In particular, the combination of in vivo and in vitro experiments using well-established incubations with human liver cell fractions is expected to give a comprehensive metabolic overview suitable for the development of toxicological screening procedures [4,14,19].

In conclusion, it is recommended to include all MS^2^ spectra of the tentatively identified metabolites in comprehensive screening libraries used for the toxicological urine screening approaches whenever possible. This is also of importance as some common metabolites of drugs may need cautious analytical interpretation. The metabolite M3 formed after demethylation of MASProp or *N,N*-bis-dealkylation of PySProp was identical to ASProp (see Figure 3, Figure 4 and Figure 6). The *N*-acetylated follow-up metabolite M2 can therefore also be formed during the metabolic transformation of different parent compounds. This is also true for M7 and M8, which were identified as metabolic pathways of MASPent as well as of PySPent (see Figure 5 and Figure 7). Nevertheless, after consumption of cathinone derivatives, the parent compounds are regularly detectable in human urine [4,25,26]. In the present study, all five parent compounds were still detectable after pHLS9 incubations and in rat urine samples. Therefore, the parent compounds are recommended to be included in urine screening procedures as well.

## 4. Conclusions

Thirty-eight metabolites of ASProp, MASProp, MASPent, PySProp, and PySPent were tentatively identified in vitro and/or in vivo. However, four metabolites (M2, M3, M7, and M8) were found to be common metabolites, calling for cautious analytical interpretation if these compounds are detected in patient biosamples. While the total number of metabolites detectable in pHLS9 incubations and rat urine precipitates was comparable (29 in pHLS9, 26 in rat urine), the latter were more suitable for the detection of phase II metabolites (1 in pHLS9, 5 in rat urine). All compounds showed metabolic reactions like those observed for other synthetic cathinones. The contribution of several isozymes to their metabolism minimizes the risk of drug–drug interactions. However, the identified metabolites should be considered as additional targets, especially in urine screening procedures, while RPLC and HILIC can both be recommended as part of the analytical setup. Furthermore, the information provided regarding these novel substances will be relevant to professionals involved in clinical and forensic analysis.

## Data Availability

The data that support the findings of this study are available from the corresponding author upon reasonable request.

## Supplementary Materials

The following supporting information can be downloaded at: https://www.mdpi.com/article/10.3390/metabo15080497/s1, Figure S1: Nuclear magnetic resonance spectroscopy data of ASProp, MASProp, MASPent, PySProp, and PySPent hydrochloride salts, Table S1: TF Q Exactive Plus Orbitrap MS conditions, Table S2: ASProp, MASProp, MASPent, PySProp, PySPent, and their phase I and II metabolites identified in in vitro and/or in vivo by means of HRMS/MS together with their identification numbers, metabolic reactions, precursor ion masses, characteristic fragment ions and relative intensities in MS^2^, calculated exact masses, elemental compositions, deviations of the measured from the calculated masses, and retention times using reversed-phase liquid chromatography or hydrophilic interaction liquid chromatography, Table S3: General involvement of monooxygenases in the formation of the given ASProp, MASProp, MASPent, PySProp, and PySPent single-step phase I metabolites.
