# Supplementary material for: A Comparative Study Using Reversed-Phase and Hydrophilic Interaction Liquid Chromatography to Investigate the In Vitro and In Vivo Metabolism of Five Selenium-Containing Cathinone Derivatives
Source: Metabolites. 2025 Jul 23;15(8):497. doi: 10.3390/metabo15080497 (PMC12388223; doi:10.3390/metabo15080497)
Supplement: Supplementary file 1 [file metabolites-15-00497-s001.zip › metabolites-3726596-supplementary.pdf]

Supplementary Materials

A comparative study using reversed-phase and hydrophilic interaction liquid chromatography to investigate the in vitro and in vivo metabolism of five selenium-containing cathinone derivatives

Lea Wagmann, Jana H. Schmitt, Tanja M. Gampfer, Simon D. Brandt, Kenneth Scott, Pierce V. Kavanagh and Markus R. Meyer

2.4. pHLS9 incubations

The final incubation volume was 150 µL obtaining a protein concentration of 2 mg/mL. All given concentrations are the concentrations in the final incubation mixture. First, a mixture containing 90 mM phosphate buffer (pH 7.4), 25 µg/mL alamethicin (UGT reaction mixture solution B), 2.5 mM $MgCl_2$, 2.5 mM isocitrate, 0.6 mM $NADP^+$, 0.8 U/mL isocitrate dehydrogenase, 100 U/mL superoxide dismutase, 0.1 mM acetyl coenzyme A, and 2 mg/mL pHLS9 was preincubated for 10 min at 37°C. Afterwards, 2.5 mM uridine 5'-diphospho-glucuronic acid (UGT reaction mix solution A), 40 µM PAPS, 1.2 mM SAM, 1 mM dithiothreitol, and 10 mM reduced glutathione were added. The addition of 25 µM of ASProp, MASProp, MASPent PySProp, PySPent, or 50 µM of ASProp in phosphate buffer started the reactions and the mixture was incubated for 360 min. After 60 min, 60 µL of the incubation mixture were transferred into another tube and the reaction was terminated by addition of 20 µL ice-cold acetonitrile containing 2.5 µM trimipramine-d3 as internal standard. The remaining mixture (90 µL) was incubated for additional 5 h and stopped with 30 µL ice-cold acetonitrile containing 2.5 µM trimipramine-d3. After cooling for 30 min at -20°C, all samples were centrifuged for 2 min at 18,407 x g, and 50 µL of the supernatants were transferred to autosampler vials. Blank incubations without selenium-containing compound and control samples without pHLS9 were prepared to confirm the absence of interfering compounds and to identify compounds that are not of metabolic origin, respectively. All incubations were done in duplicates.

2.5. Rat urine collection and sample preparation

Male Wistar rats (Charles River, Sulzfeld, Germany) were used for the metabolism studies. The animal experiments were in accordance with the German law for animal protection and have been approved by an ethics committee (Landesamt für Verbraucherschutz, Saarbrücken, Germany). Each rat (weight 250 - 270 g) was treated with one of the compounds included in this study. Before compound administration, blank urine was collected to confirm the absence of interfering compounds. The compounds were orally administered in an aqueous suspension by gastric intubation in doses of 1 mg/kg body weight. After administration, the rats were housed in metabolism cages for 24 h. Rats had water ad libitum during the collection of urine, which was caught separately from feces. Sample preparation was performed by adding 0.5 mL of acetonitrile to 0.1 mL of rat urine. The mixture was shaken on a rotary shaker for 2 min at 2,000 rpm. After centrifugation for 3 min at 18,407 × g, 0.5 mL was transferred into a glass vial and evaporated to dryness under a gentle stream of nitrogen at 70°C. The residue was dissolved in 50 µL of a mixture of water:acetonitrile (1:1; v/v) containing 0.1% formic acid and 5 mM ammonium formate before analysis.

2.6. Monooxygenases activity screening

The microsomal incubations were performed at 37°C for 30 min for ASProp, MASProp, MASPent, PySProp, PySPent, or 60 min for ASProp (25 µM, each) with 50 pmol/mL of each CYP isoenzyme or 0.25 mg protein/mL FMO3. All given concentrations are final concentrations in the incubation mixture. The incubation mixtures with a final volume of 50 µL also contained 90 mM phosphate buffer (pH 7.4), 1.2 mM $NADP^+$, the $NADP^+$ regenerating system (5 mM $MgCl_2$, 5 mM isocitrate, 0.5 U/mL isocitrate dehydrogenase), and 200 U/mL superoxide dismutase. All incubations were performed with phosphate buffer except for the ones with CYP2A6 and CYP2C9 which were performed with 90 mM Tris buffer (pH 7.4) in accordance to the manufacturer's recommendations. A positive control containing 1 mg protein/mL pHLM and a blank incubation not containing any metabolizing enzyme was also performed. The addition of $NADP^+$ and the $NADP^+$ regenerating system started the reactions, which were finally terminated by addition of 50 µL of ice-cold acetonitrile containing 2.5 µM trimipramine-d3 as internal standard. was All samples were centrifuged for 5 min at 18,407 x g and 50 µL of the supernatant were transferred to an autosampler vial. All incubations were done in duplicates.

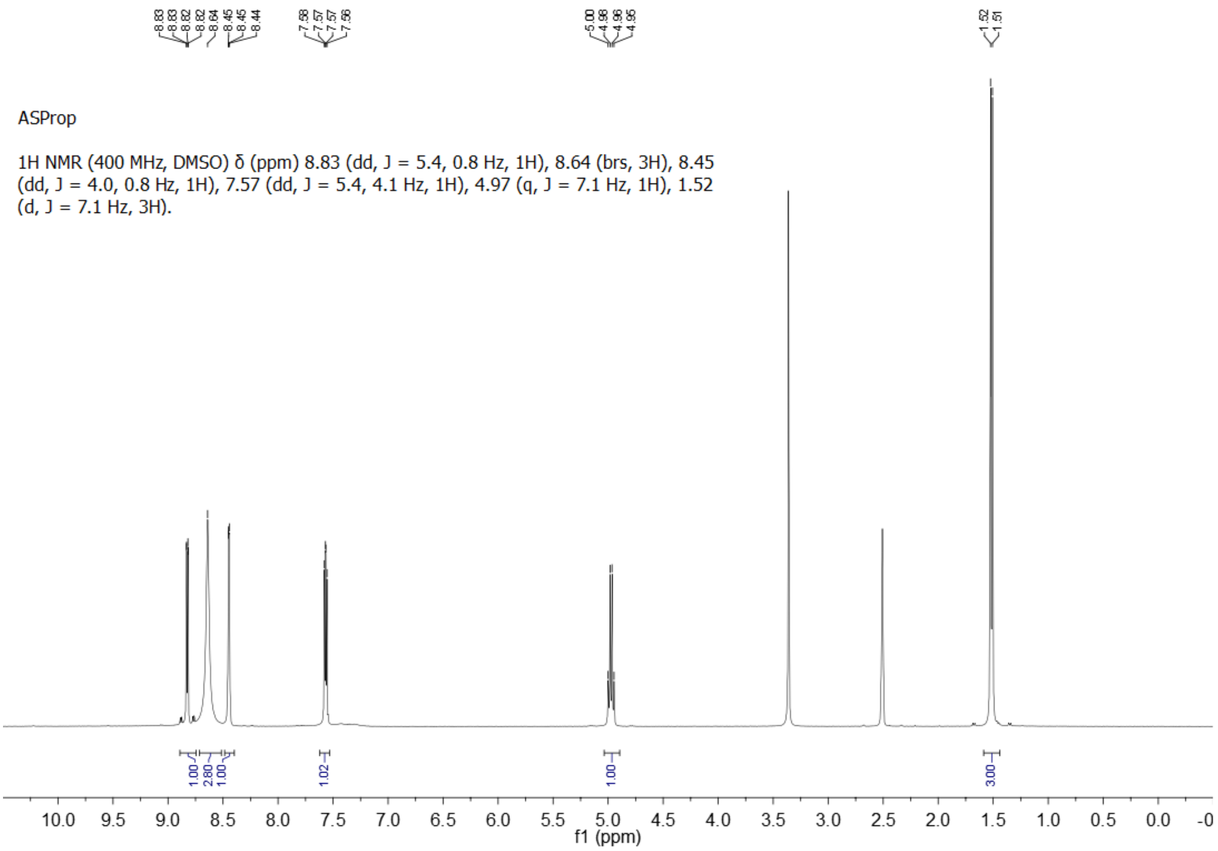

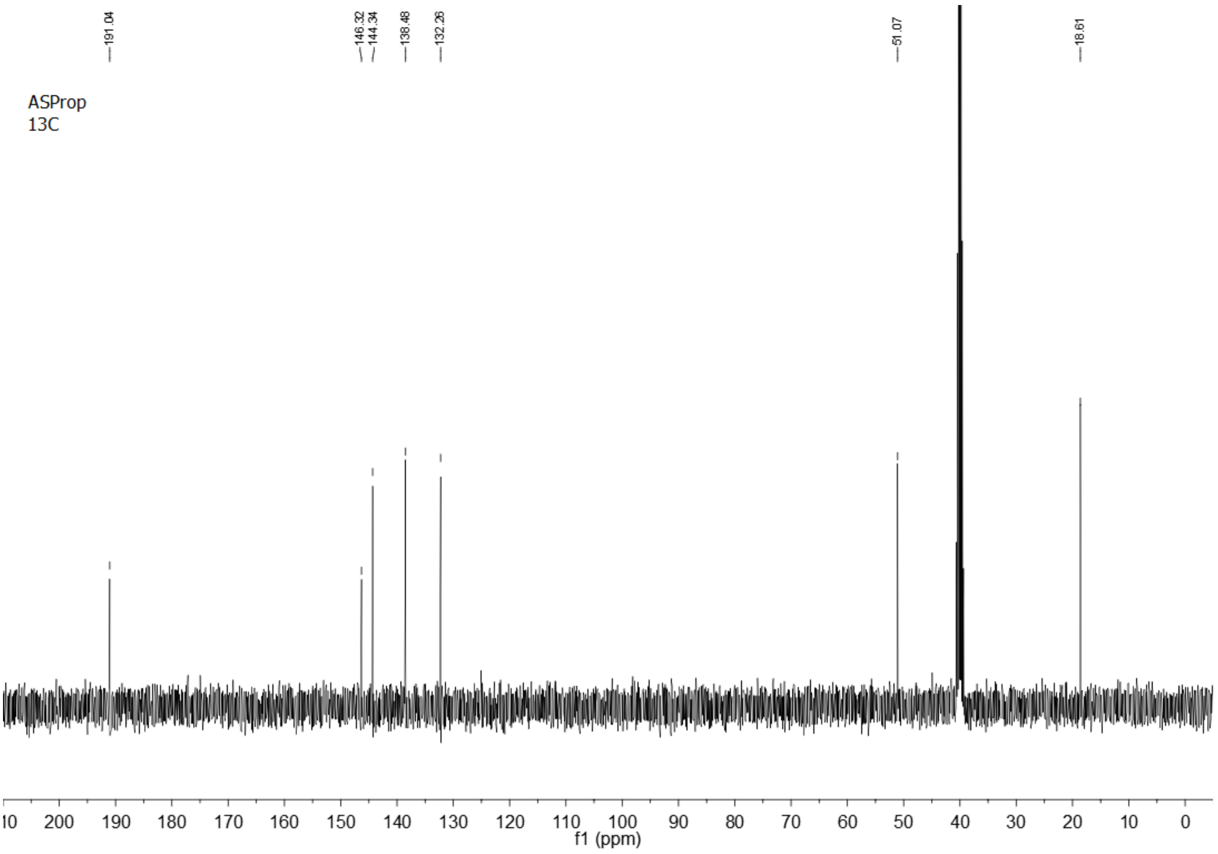

Figure S1: Nuclear magnetic resonance spectroscopy data of ASProp, MASProp, MASPent, PySProp, and PySPent hydrochloride salts.

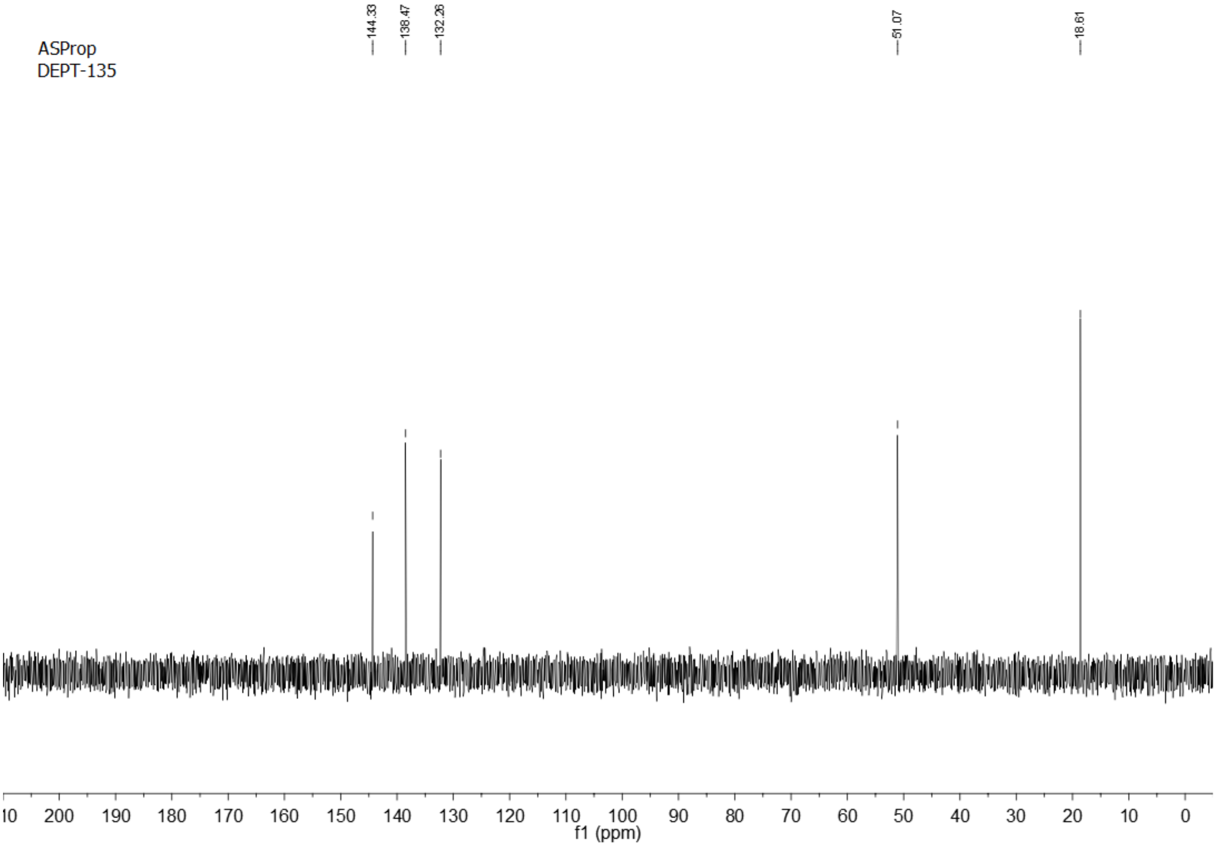

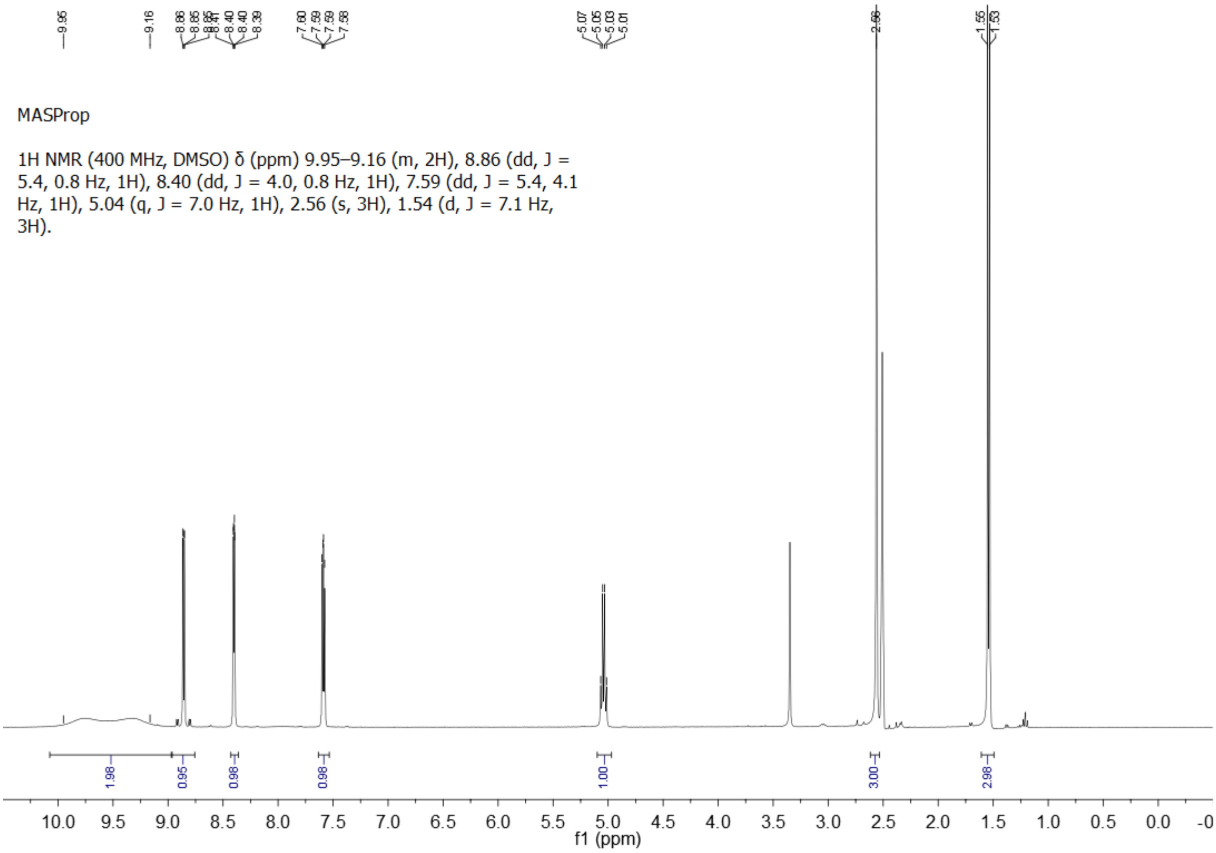

Figure S1: continued

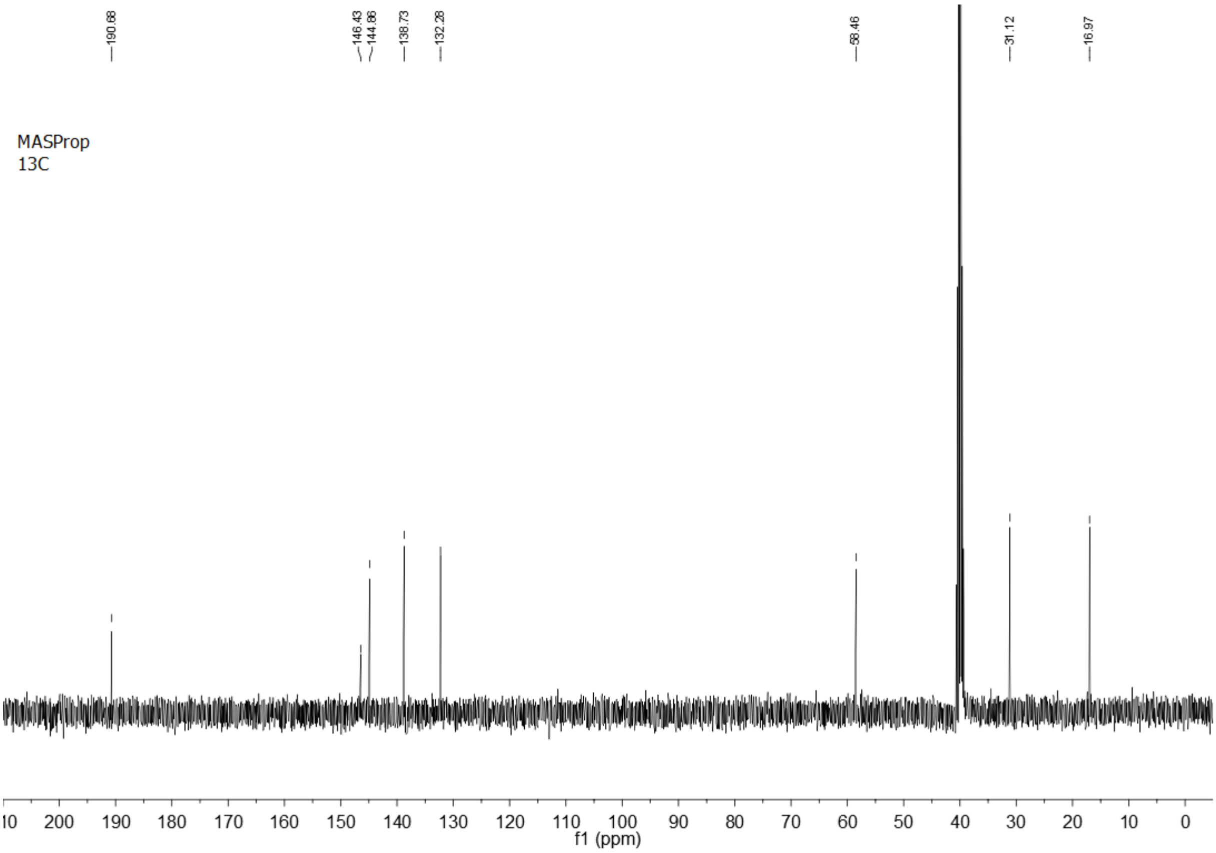

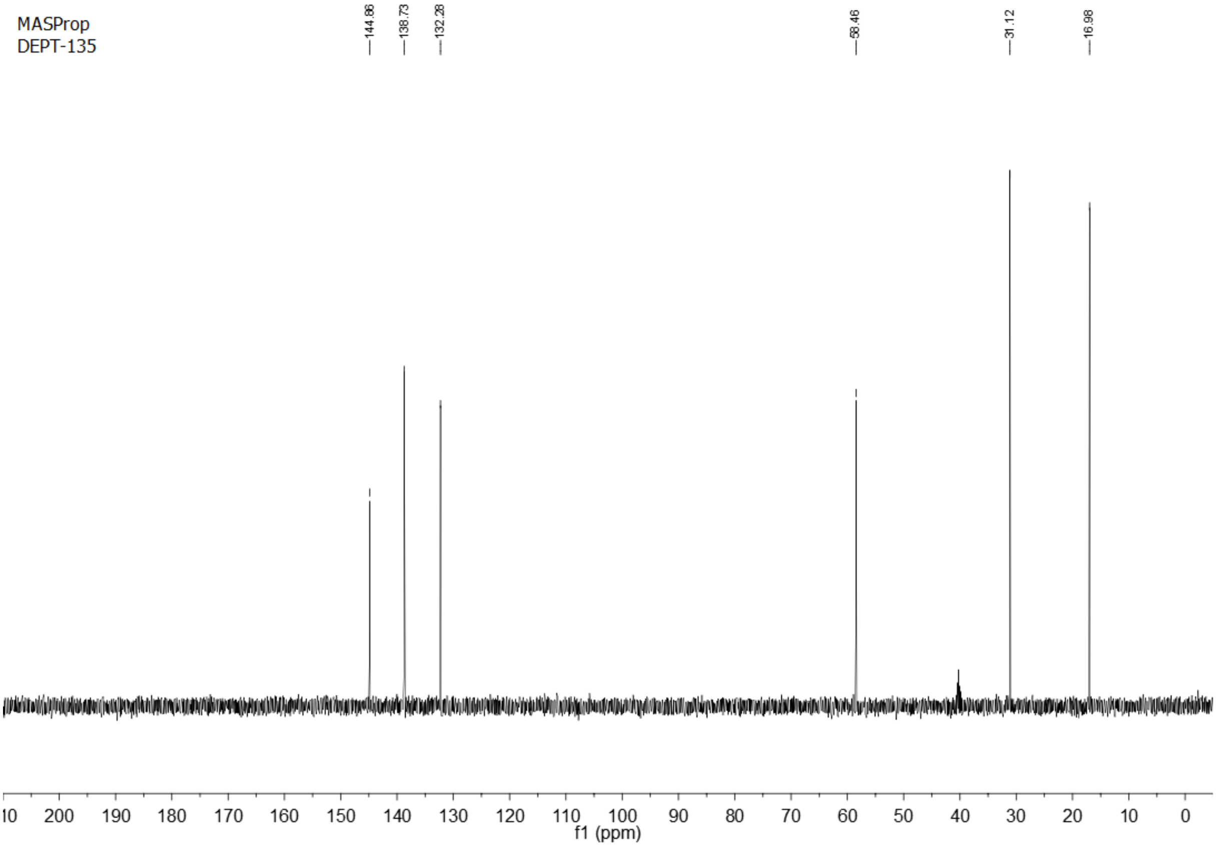

Figure S1: continued

MASPent

1H NMR (400 MHz, DMSO) δ (ppm) 10.12–9.06 (m, 2H), 8.87 (dd, J = 5.4, 0.8 Hz, 1H), 8.44 (dd, J = 4.0, 0.9 Hz, 1H), 7.59 (dd, J = 5.4, 4.1 Hz, 1H), 5.08 (t, J = 5.7 Hz, 1H), 2.53 (s, 3H), 2.00–1.86 (m, 2H), 1.38–1.13 (m, 2H), 0.84 (t, J = 7.3 Hz, 3H).

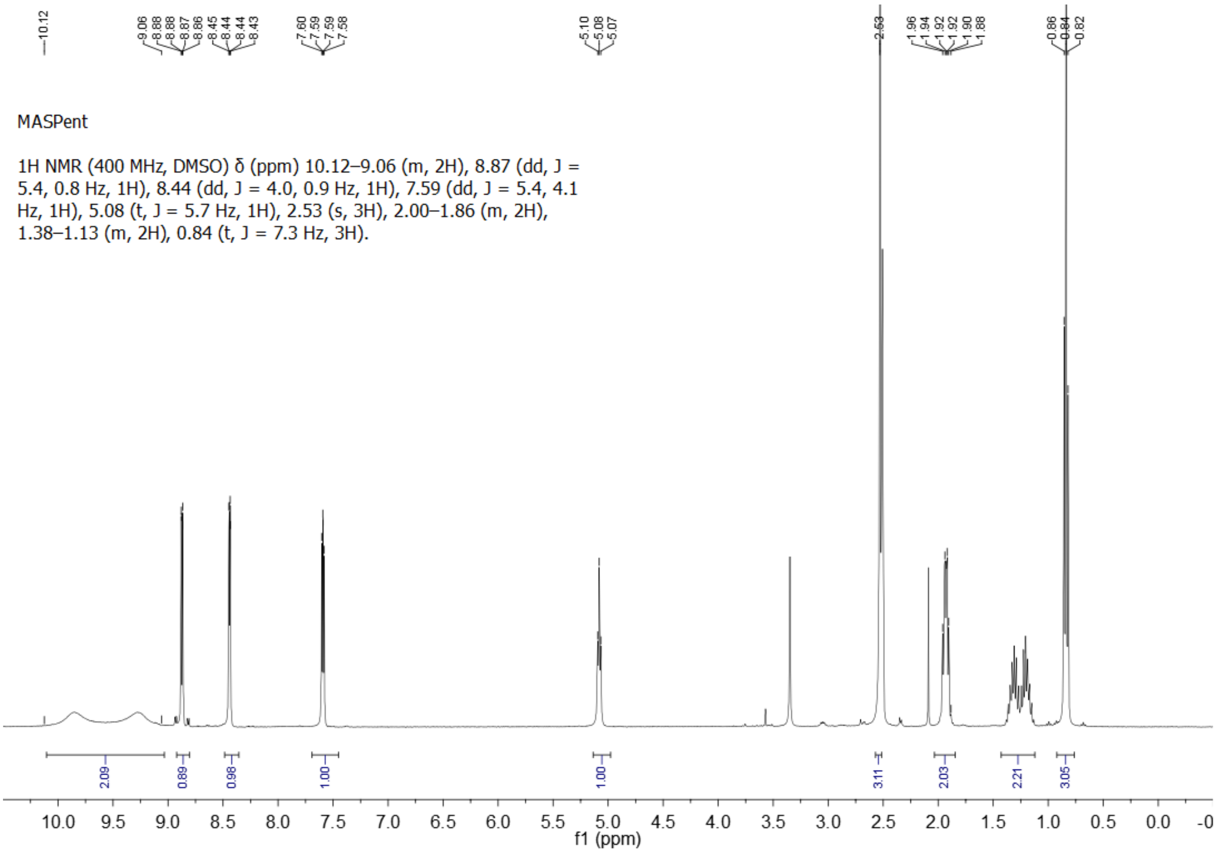

MASPent
13C

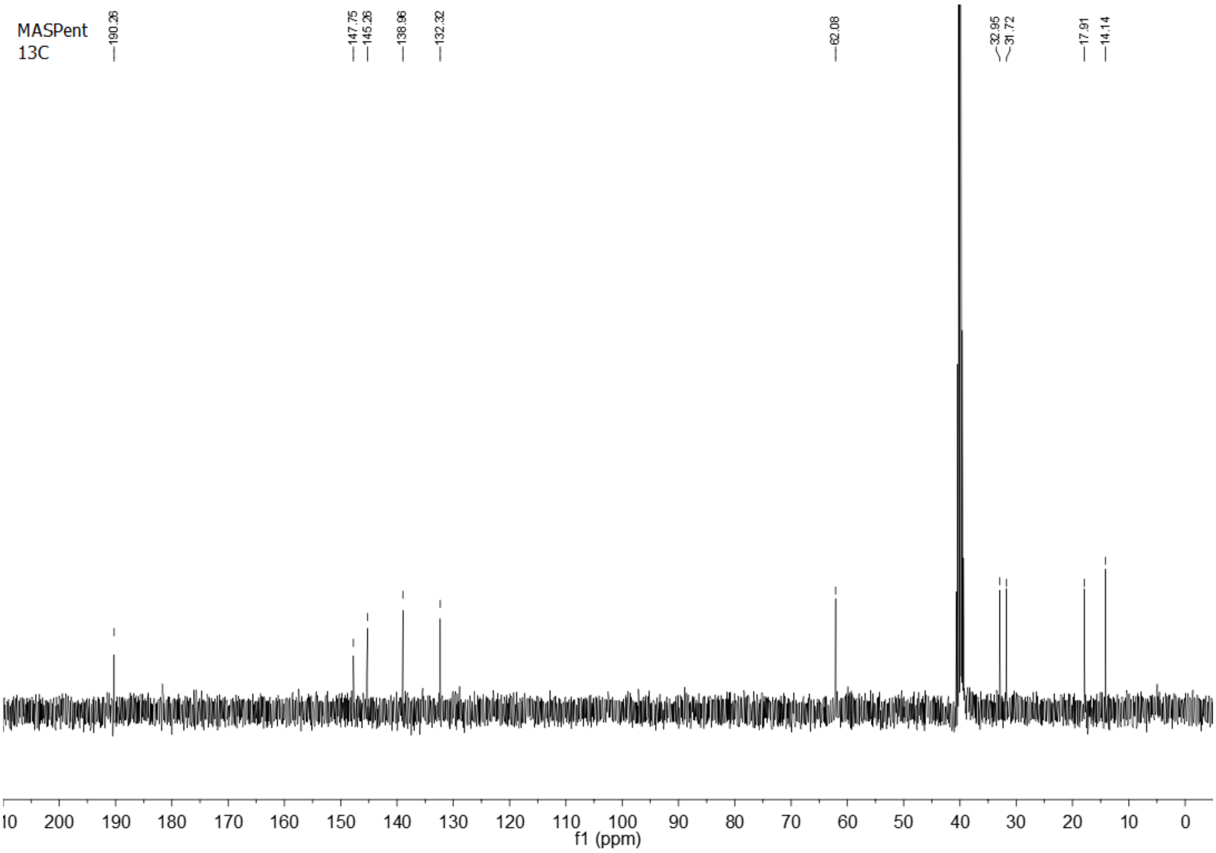

Figure S1: continued

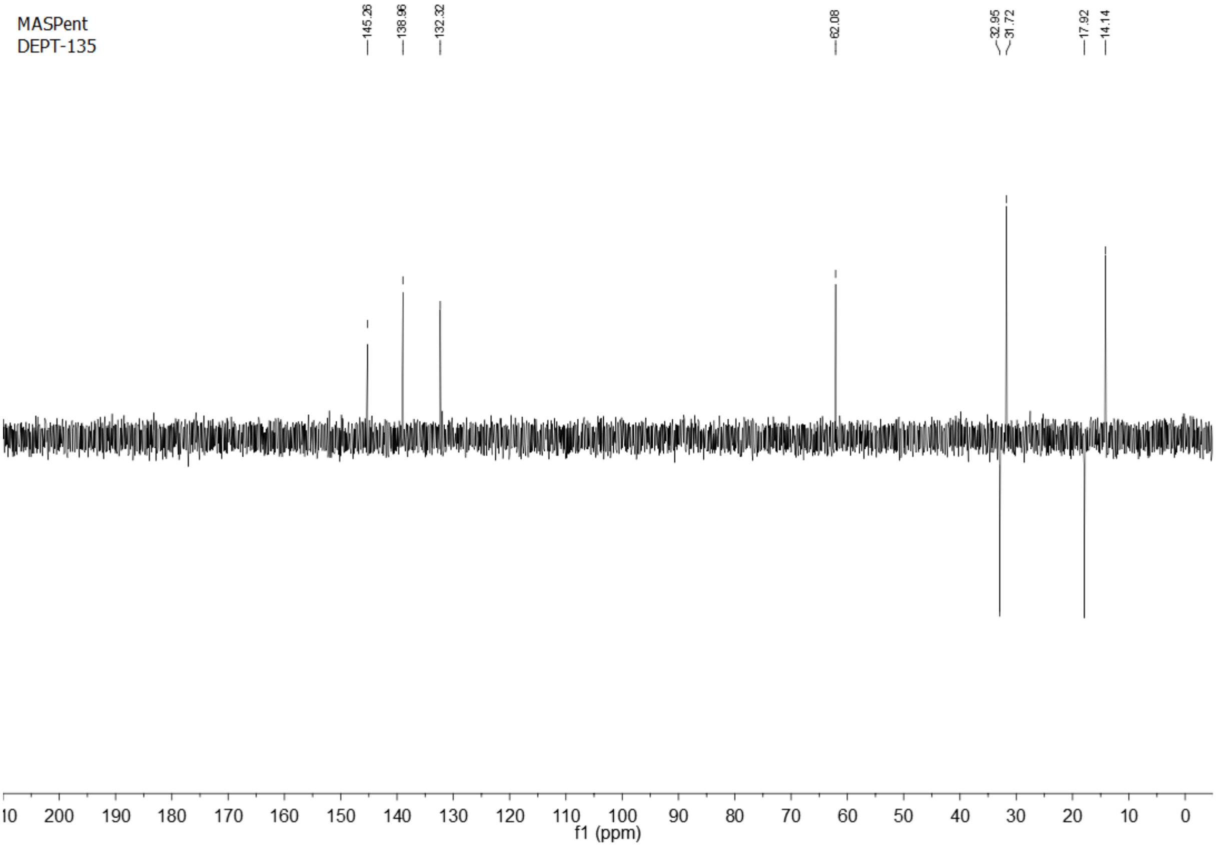

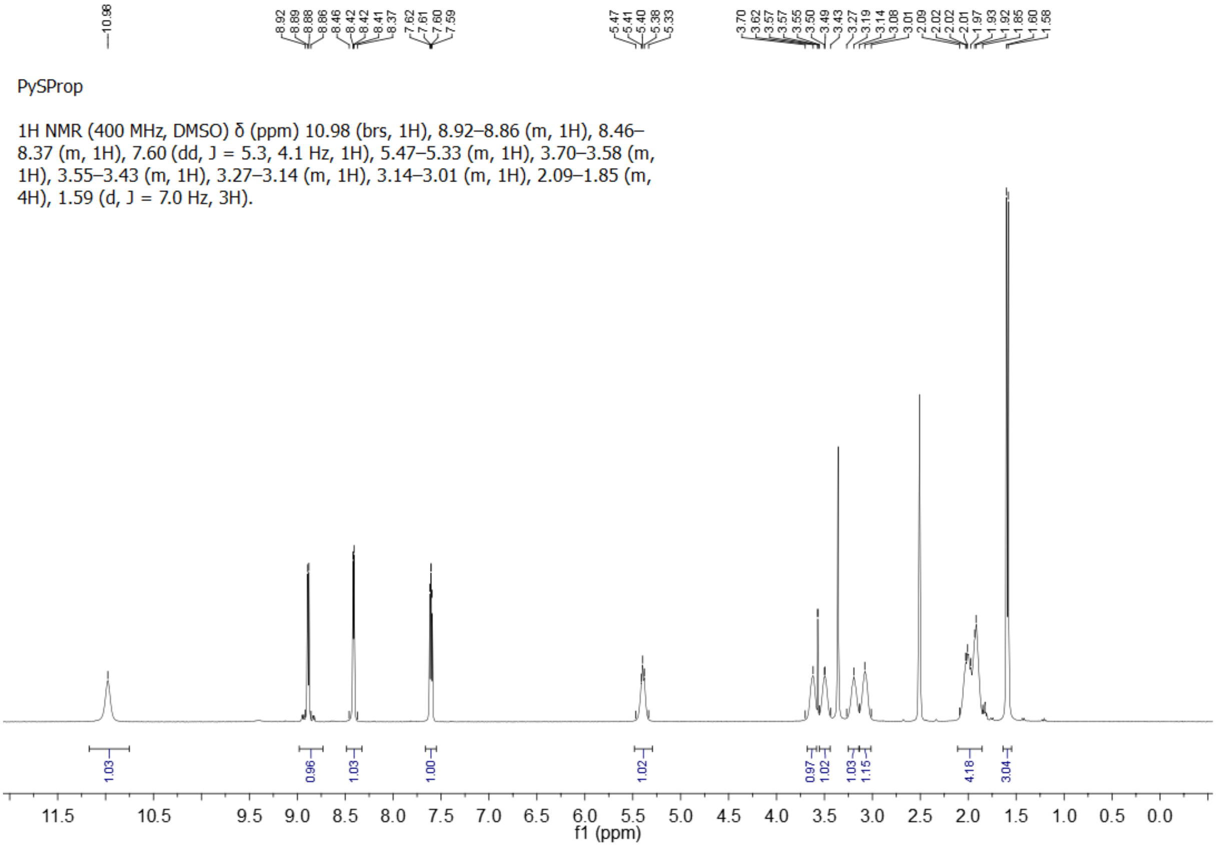

Figure S1: continued

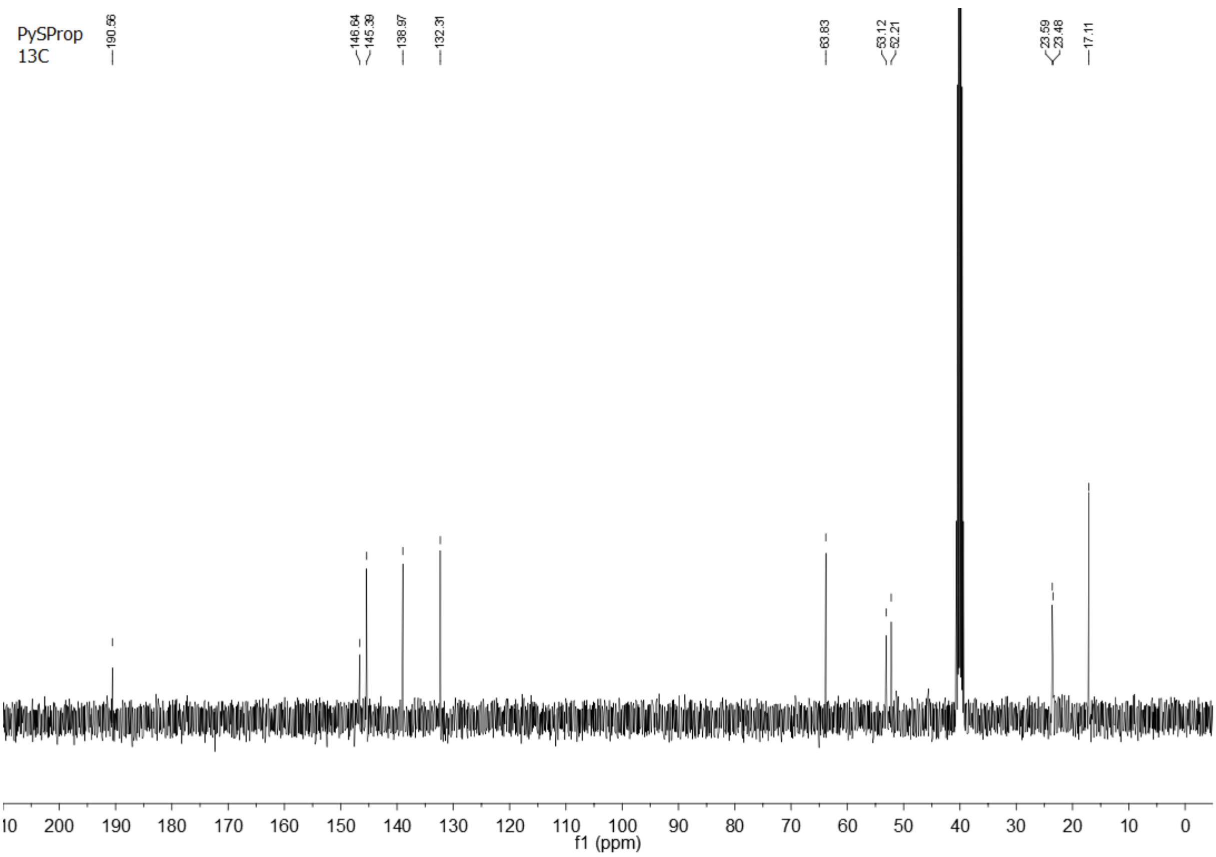

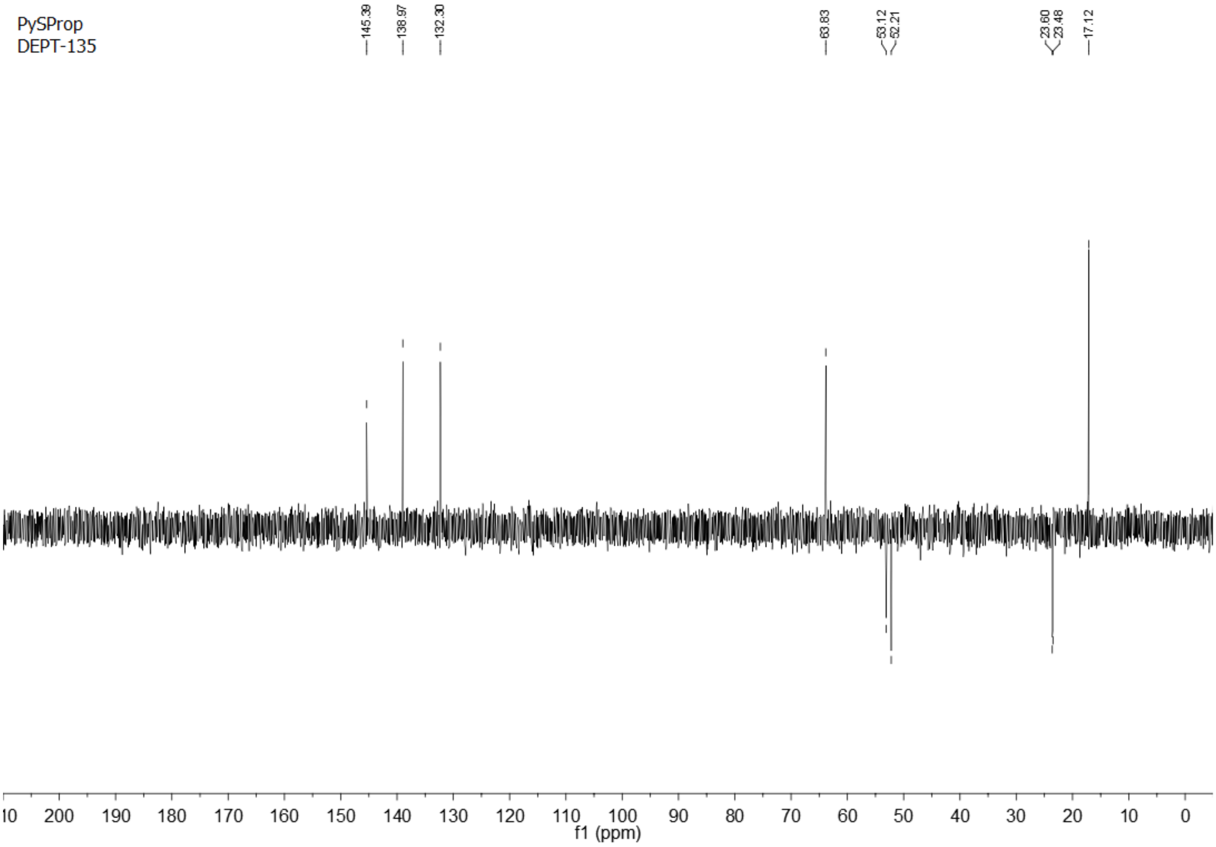

Figure S1: continued

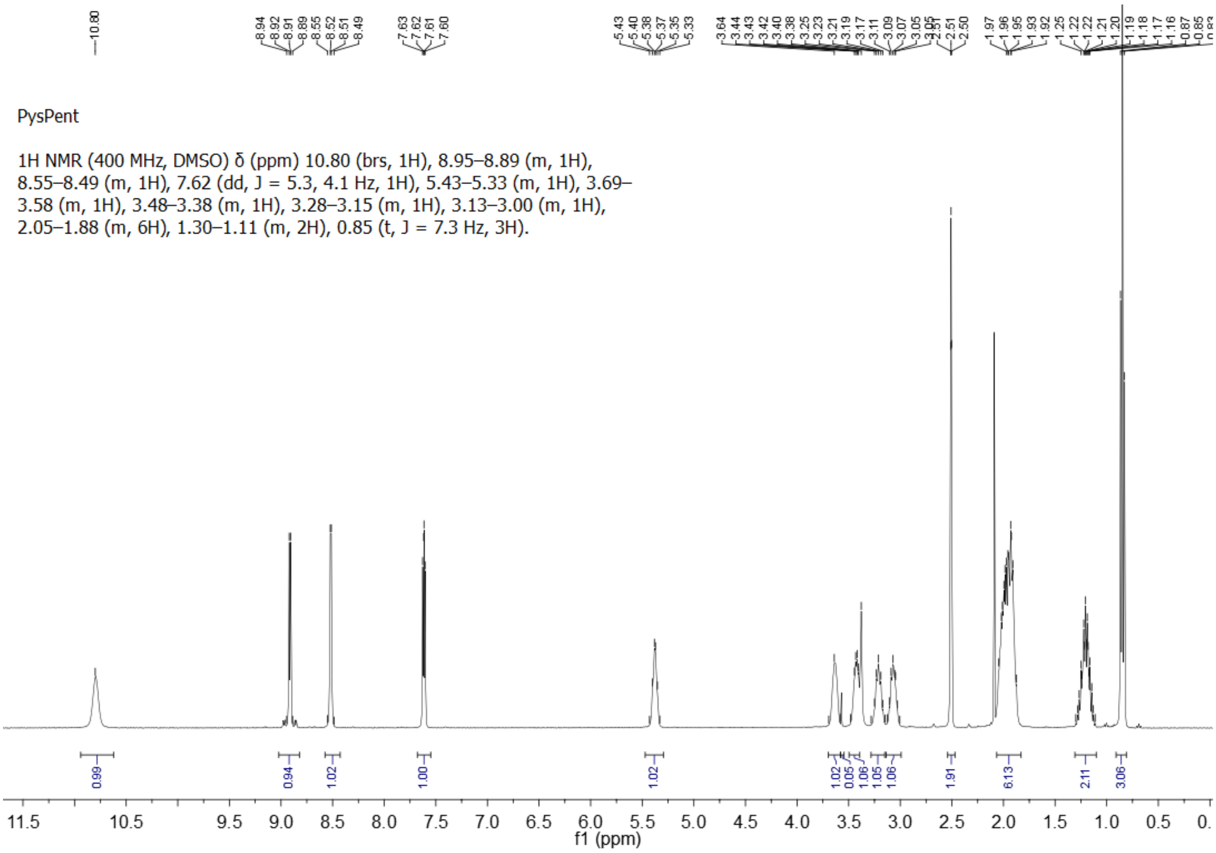

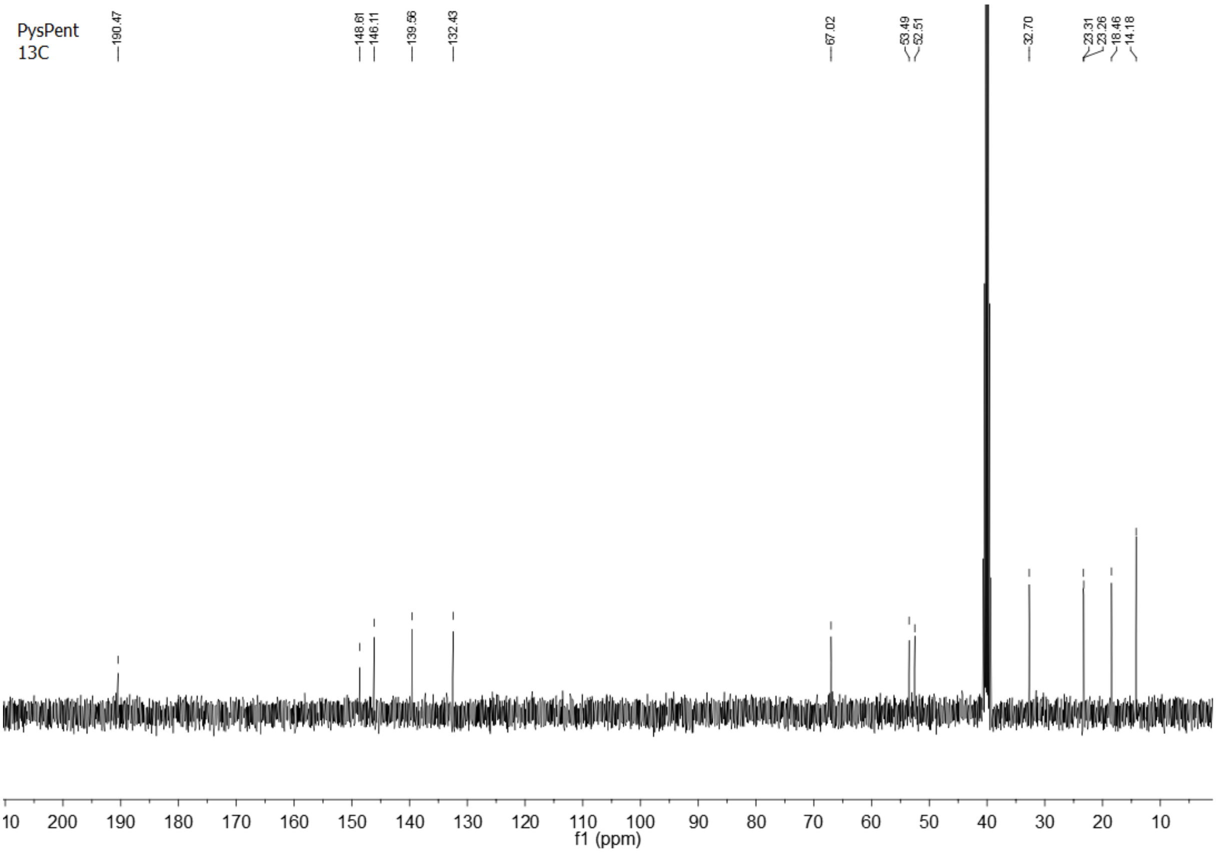

Figure S1: continued

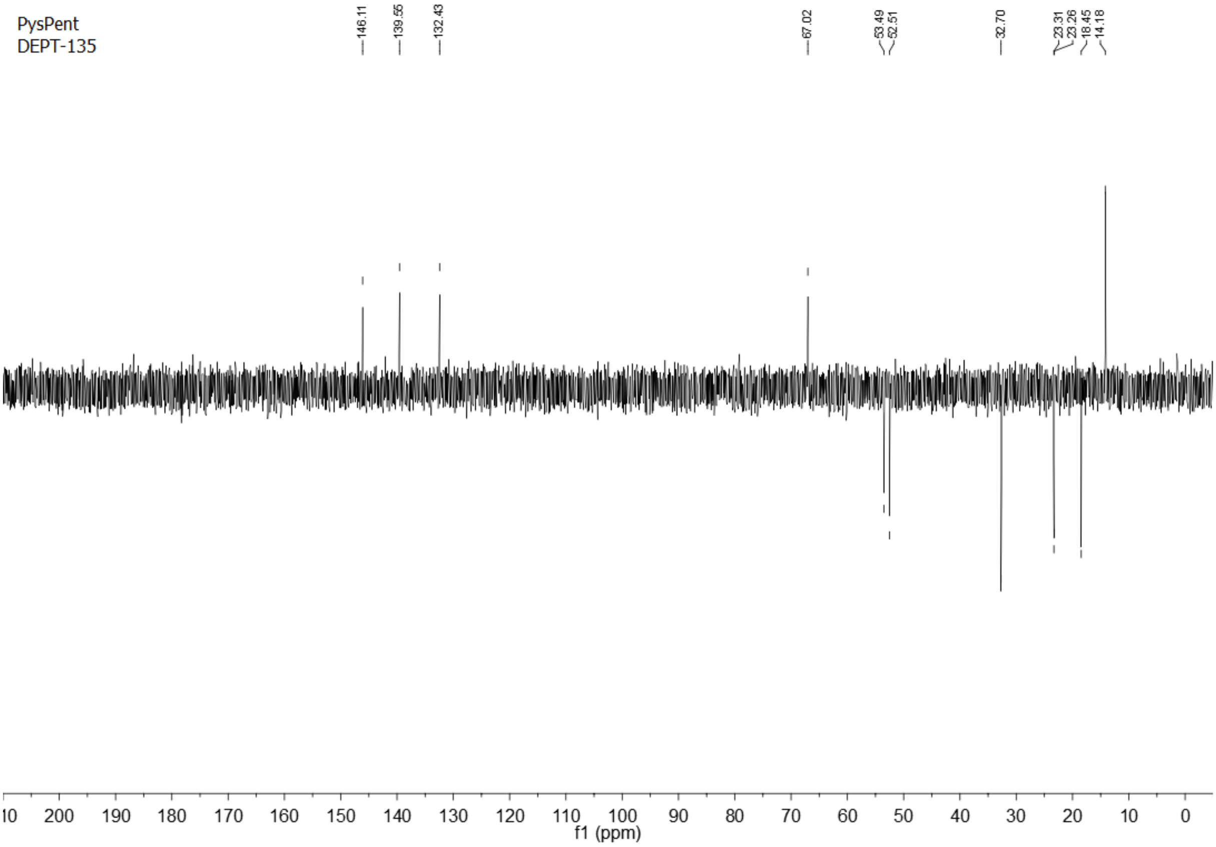

Figure S1: continued

Table S1: TF Q Exactive Plus Orbitrap MS conditions

**Parameter**	**Settings**
Ionization mode	Positive
Sheath gas	60 arbitrary units
Auxiliary gas	10 arbitrary units
Spray voltage	4.0 kV
Heater temperature	320 °C
Ion transfer capillary temperature	320 °C
S-lens RF level	50.0
Inclusion list	On
Full scan data acquisition	
Resolution	35,000
Scan range	100-600 *m/z*
Automatic gain control	1e6
Maximum injection time	120 ms
Data-dependent MS^2	
Resolution	17,500
Isolation window	*m/z* 1.0
AGC target	2e5
Maximum injection time	250 ms
Stepped normalized collision energy	17.5 %, 35.0 %, 52.5 %
Loop count	5
Pick others	Enabled
Exclude isotopes	On
Spectrum data type	Profile

Table S2. ASProp, MASProp, MASPent, PySProp, PySPent, and their phase I and II metabolites identified in in vitro incubations with pooled human liver S9 fraction (pHLS9) and/or rat urine (RU) by means of HRMS/MS together with their identification numbers (ID), metabolic reactions, precursor ion masses (PM) recorded in MS^1 in positive mode, characteristic fragment ions (FI) in MS^2, relative intensities in MS^2, calculated exact masses, elemental compositions, deviations of the measured from the calculated masses, and retention times (RT) using reversed-phase liquid chromatography (RPLC) or hydrophilic interaction liquid chromatography (HILIC).

Metabolite ID	Metabolic reaction	Detected in	Elemental composition	Characteristic ions at measured accurate masses, *m/z*	Relative intensity in MS^2, %	Calculated exact masses, *m/z*	Error, ppm	RT RPLC, min	RT HILIC, min
**ASProp**	Parent compound	pHLS9, RU	$C_7H_{10}ONSe$	PM at *m/z* 203.9920	10	203.9922	-1.1	2.2	5.3
			C_7H_7OSe	FI at *m/z* 186.9655	8	186.9657	-0.7		
			C_7H_8NSe	FI at *m/z* 185.9816	100	185.9816	-0.1		
			C_6H_7Se	FI at *m/z* 158.9706	13	158.9707	-0.7		
			C_7H_7N	FI at *m/z* 105.0576	16	105.0573	3.2		
			C_6H_8N	FI at *m/z* 94.0656	21	94.0651	5.2		
M1	Reduction	pHLS9, RU	$C_7H_{12}ONSe$	PM at *m/z* 206.0085	5	206.0079	3.3	1.7	5.8
			$C_7H_{10}NSe$	FI at *m/z* 187.9974	100	187.9973	0.7		
			C_5H_7Se	FI at *m/z* 146.9708	37	146.9707	0.4		
			C_7H_9N	FI at *m/z* 107.0733	17	107.0730	3.6		
M2	*N*-Acetylation	RU	$C_9H_{12}O_2NSe$	PM at *m/z* 246.0025	18	246.0028	-1.2	6.1	0.9
			$C_7H_{10}ONSe$	FI at *m/z* 203.9923	86	203.9922	0.4		
			C_7H_8NSe	FI at *m/z* 185.9817	100	185.9816	0.3		
			C_6H_7Se	FI at *m/z* 158.9708	15	158.9707	0.6		
			C_6H_8N	FI at *m/z* 94.0658	9	94.0651	6.6		
**MASProp**	Parent compound	pHLS9, RU	$C_8H_{12}ONSe$	PM at *m/z* 218.0078	32	218.0078	-0.2	3.2	4.3
			$C_8H_{10}NSe$	FI at *m/z* 199.9973	100	199.9972	0.5		
			C_6H_7Se	FI at *m/z* 158.9707	15	158.9707	-0.1		
			C_8H_9N	FI at *m/z* 119.0732	20	119.0730	2.0		
			C_3H_8N	FI at *m/z* 58.0661	86	58.0651	16		
M3	*N*-Demethylation	pHLS9, RU	$C_7H_{10}ONSe$	PM at *m/z* 203.9921	11	203.9922	-0.6	2.2	5.3
			C_7H_8NSe	FI at *m/z* 185.9815	100	185.9816	-0.8		

			C_6H_7Se	FI at *m/z* 158.9706	12	158.9707	-1.0		
			C_6H_8N	FI at *m/z* 94.0656	21	94.0651	4.8		
M4	Reduction	pHLS9, RU	$C_8H_{14}ONSe$	PM at *m/z* 220.0241	4	220.0235	2.6	2.6	5.2
			$C_8H_{12}NSe$	FI at *m/z* 202.0128	100	202.0129	-0.6		
			C_7H_9NSe	FI at *m/z* 186.9890	4	186.9895	-2.5		
			C_5H_5Se	FI at *m/z* 144.9550	4	144.9550	-0.8		
			C_6H_9N	FI at *m/z* 95.0734	5	95.0730	4.9		
M5	Hydroxylation	RU	$C_8H_{12}O_2NSe$	PM at *m/z* 234.0027	11	234.0028	-0.4	1.2	5.6
			$C_8H_{10}ONSe$	FI at *m/z* 215.9922	1	215.9922	0.0		
			$C_7H_{10}NSe$	FI at *m/z* 187.9972	2	187.9973	-0.4		
			C_6H_7OSe	FI at *m/z* 174.9656	2	174.9657	-0.5		
			C_8H_9ON	FI at *m/z* 135.0680	6	135.0679	0.7		
			C_3H_8N	FI at *m/z* 58.0661	100	58.0651	16		
M6	*N*-Oxygenation	pHLS9, RU	$C_8H_{12}O_2NSe$	PM at *m/z* 234.0028	16	234.0028	0.2	5.4	0.8
			C_5H_3OSe	FI at *m/z* 158.9343	100	158.9343	-0.2		
			C_3H_8ON	FI at *m/z* 74.0608	50	74.0600	10		
M2	*N*-Demethylation & *N*-acetylation	RU	$C_9H_{12}O_2NSe$	PM at *m/z* 246.0033	18	246.0028	-1.2	6.1	0.9
			$C_7H_{10}ONSe$	FI at *m/z* 203.9923	86	203.9922	0.4		
			C_7H_8NSe	FI at *m/z* 185.9817	100	185.9816	0.3		
			C_6H_7Se	FI at *m/z* 158.9711	15	158.9707	2.3		
			C_6H_8N	FI at *m/z* 94.0657	9	94.0651	5.9		
**MASPent**	Parent compound	pHLS9, RU	$C_{10}H_{16}ONSe$	PM at *m/z* 246.0394	26	246.0391	1.2	5.5	2.5
			$C_{10}H_{14}NSe$	FI at *m/z* 228.0288	42	228.0285	1.3		
			C_7H_8NSe	FI at *m/z* 185.9819	11	185.9816	1.6		
			C_5H_5Se	FI at *m/z* 144.9553	12	144.9550	2.1		
			$C_5H_{12}N$	FI at *m/z* 86.0972	100	86.0964	9.3		
M7	*N*-Demethylation	pHLS9, RU	$C_9H_{14}ONSe$	PM at *m/z* 232.0236	12	232.0234	0.9	5.3	4.7
			$C_9H_{11}OSe$	FI at *m/z* 214.9969	11	214.9969	-0.3		
			$C_9H_{12}NSe$	FI at *m/z* 214.0131	82	214.0129	0.9		
			C_6H_6NSe	FI at *m/z* 171.9661	25	171.9659	1.2		
			C_5H_5Se	FI at *m/z* 144.9552	23	144.9550	1.4		
			$C_4H_{10}N$	FI at *m/z* 72.0817	100	72.0807	14		

M8	*N*-Demethylation & *N*-oxygenation	RU	$C_9H_{14}O_2NSe$	PM at *m/z* 248.0183	10	248.0184	-0.3	6.7	0.9
			$C_9H_{12}ONSe$	FI at *m/z* 230.0079	10	230.0079	0.1		
			C_5H_3OSe	FI at *m/z* 158.9344	12	158.9343	0.3		
			$C_4H_{10}ON$	FI at *m/z* 88.0763	100	88.0757	7.1		
M9	Reduction	pHLS9	$C_{10}H_{18}ONSe$	PM at *m/z* 248.0554	5	248.0547	2.6	5.4	4.6
			$C_{10}H_{16}NSe$	FI at *m/z* 230.0444	100	230.0442	0.9		
			$C_9H_{11}Se$	FI at *m/z* 199.0022	6	146.9707	0.5		
			C_7H_9NSe	FI at *m/z* 186.9896	16	132.9551	0.5		
			$C_5H_{10}N$	FI at *m/z* 84.0815	38	86.0964	8.6		
M10	Hydroxylation	pHLS9	$C_{10}H_{16}O_2NSe$	PM at *m/z* 262.0341	10	262.0340	0.4	4.3	5.1
			$C_5H_3O_2Se$	FI at *m/z* 174.9299	1	174.9292	4.0		
			$C_{10}H_{13}ON$	FI at *m/z* 163.0996	1	163.0991	3.1		
			C_5H_5OSe	FI at *m/z* 160.9503	1	160.9500	2.5		
			C_4H_3OSe	FI at *m/z* 146.9342	1	146.9343	-0.7		
			$C_5H_{12}N$	FI at *m/z* 86.0972	100	86.0964	9.3		
M11	Hydroxylation	pHLS9	$C_{10}H_{16}O_2NSe$	PM at *m/z* 262.0345	14	262.0340	1.4	5.0	1.1
			$C_5H_3O_2Se$	FI at *m/z* 174.9294	2	174.9292	0.7		
			C_5H_5OSe	FI at *m/z* 160.9501	2	160.9500	0.3		
			$C_5H_{12}N$	FI at *m/z* 86.0972	100	86.0964	9.0		
M12	*N*-Oxygenation	pHLS9	$C_{10}H_{16}O_2NSe$	PM at *m/z* 262.0341	23	262.0340	0.4	7.6	0.8
			$C_{10}H_{14}ONSe$	FI at *m/z* 244.0247	2	244.0235	4.7		
			C_5H_3OSe	FI at *m/z* 158.9346	100	158.9343	1.9		
			C_5H_5Se	FI at *m/z* 144.9556	5	144.9550	4.1		
			$C_5H_{12}ON$	FI at *m/z* 102.0920	68	102.0913	6.7		
**PySProp**	Parent compound	pHLS9, RU	$C_{11}H_{16}ONSe$	PM at *m/z* 258.0391	70	258.0392	-0.2	4.5	1.2
			C_7H_7OSe	FI at *m/z* 186.9656	11	186.9657	-0.3		
			C_6H_7Se	FI at *m/z* 158.9706	10	158.9707	-0.7		
			$C_7H_{12}ON$	FI at *m/z* 126.0915	1	126.0913	1.4		
			$C_6H_{12}N$	FI at *m/z* 98.0970	100	98.0964	5.4		
			C_4H_8N	FI at *m/z* 70.0659	4	70.0651	11		
M3	*N,N*-bis-Dealkylation	pHLS9, RU	$C_7H_{10}ONSe$	PM at *m/z* 203.9923	11	203.9922	0.3	2.2	5.3
			C_7H_8NSe	FI at *m/z* 185.9816	100	185.9816	-0.5		

			C_6H_7Se	FI at *m/z* 158.9706	12	158.9707	-1.2		
			C_6H_8N	FI at *m/z* 94.0656	21	94.0651	5.2		
M13	Reduction	pHLS9	$C_{11}H_{18}ONSe$	PM at *m/z* 260.0552	12	260.0548	1.5	4.6	1.6
			$C_{11}H_{16}NSe$	FI at *m/z* 242.0441	100	242.0442	-0.4		
			$C_{10}H_{13}NSe$	FI at *m/z* 227.0206	12	227.0208	-0.8		
			C_7H_9Se	FI at *m/z* 172.9864	17	172.9864	0.1		
			$C_6H_{12}N$	FI at *m/z* 98.0969	15	98.0964	4.9		
			C_4H_8N	FI at *m/z* 70.0659	17	70.0651	11		
M14	Hydroxylation & oxidation	RU	$C_{11}H_{14}O_2NSe$	PM at *m/z* 272.0177	22	272.0184	-2.8	4.3	5.4
			$C_{11}H_{12}ONSe$	FI at *m/z* 254.0078	18	254.0079	-0.3		
			C_7H_8NSe	FI at *m/z* 185.9816	17	185.9816	-0.1		
			$C_7H_{10}O_2N$	FI at *m/z* 140.0706	8	140.0706	0.0		
			$C_6H_{10}ON$	FI at *m/z* 112.0760	100	112.0757	2.9		
			C_4H_5O	FI at *m/z* 69.0343	5	69.0335	11		
M15	Hydroxylation & oxidation	pHLS9, RU	$C_{11}H_{14}O_2NSe$	PM at *m/z* 272.0192	15	272.0184	3.0	7.8	0.8
			$C_7H_{10}O_2N$	FI at *m/z* 140.0711	9	140.0706	3.6		
			$C_6H_{10}ON$	FI at *m/z* 112.0765	100	112.0757	6.9		
			C_4H_5O	FI at *m/z* 69.0346	5	69.0335	16		
M16	Hydroxylation	pHLS9	$C_{11}H_{16}O_2NSe$	PM at *m/z* 274.0338	36	274.0341	-0.7	2.7	4.5
			$C_{11}H_{14}ONSe$	FI at *m/z* 256.0233	43	256.0235	-0.4		
			C_5H_3OSe	FI at *m/z* 158.9344	100	158.9344	0.6		
			$C_6H_{12}ON$	FI at *m/z* 114.0916	8	114.0913	2.5		
			$C_6H_{10}N$	FI at *m/z* 96.0813	11	96.0808	6.2		
M17	Hydroxylation	pHLS9, RU	$C_{11}H_{16}O_2NSe$	PM at *m/z* 274.0347	41	274.0341	2.3	3.6	2.8
			$C_7H_7O_2Se$	FI at *m/z* 202.9610	1	202.9606	-0.4		
			C_6H_7OSe	FI at *m/z* 174.9663	3	174.9657	3.4		
			$C_6H_{12}N$	FI at *m/z* 98.0973	100	98.0964	8.7		
			$C_4H_{10}N$	FI at *m/z* 72.0818	5	72.0808			
M18	Hydroxylation	pHLS9, RU	$C_{11}H_{16}O_2NSe$	PM at *m/z* 274.0347	100	274.0341	2.4	3.9	2.2
			$C_{10}H_{14}NSe$	FI at *m/z* 228.0294	8	228.0286	3.7		
			C_6H_7Se	FI at *m/z* 158.9712	13	158.9707	3.0		
			$C_6H_{12}ON$	FI at *m/z* 114.0920	81	114.0913	5.7		

			$C_6H_{10}N$	FI at *m/z* 96.0816	4	96.0808	8.9		
M19	Hydroxylation	pHLS9	$C_{11}H_{16}O_2NSe$	PM at *m/z* 274.0334	60	274.0341	-2.3	4.7	1.8
			$C_{11}H_{14}ONSe$	FI at *m/z* 256.0232	61	256.0235	-1.4		
			$C_{11}H_{13}ON$	FI at *m/z* 175.0991	23	175.0992	-0.5		
			C_5H_3OSe	FI at *m/z* 158.9343	100	158.9344	-0.4		
			$C_6H_{12}ON$	FI at *m/z* 114.0916	15	114.0913	1.9		
			$C_6H_{10}N$	FI at *m/z* 96.0812	11	96.0808	4.7		
M20	*N*-Oxygenation	pHLS9, RU	$C_{11}H_{16}O_2NSe$	PM at *m/z* 274.0337	100	274.0341	-1.2	5.2	2.0
			C_5H_3OSe	FI at *m/z* 158.9356	13	158.9344	7.6		
			$C_6H_{12}N$	FI at *m/z* 98.0969	80	98.0964	4.6		
			C_4H_8ON	FI at *m/z* 86.0607	61	86.0600	7.3		
M21	Dihydroxylation	pHLS9, RU	$C_{11}H_{16}O_3NSe$	PM at *m/z* 290.0286	83	290.0290	-1.3	4.3	5.3
			$C_{11}H_{14}O_2NSe$	FI at *m/z* 272.0178	27	272.0184	-2.3		
			$C_{11}H_{12}ONSe$	FI at *m/z* 254.0074	13	254.0079	-1.9		
			C_7H_8NSe	FI at *m/z* 185.9814	19	185.9816	-1.6		
			$C_6H_{12}O_2N$	FI at *m/z* 130.0862	23	130.0863	-0.4		
			$C_6H_{10}ON$	FI at *m/z* 112.0760	100	112.0757	2.8		
			$C_4H_7O_2$	FI at *m/z* 87.0447	33	87.0441	7.0		
M22	Hydroxylation + glucuronidation	RU	$C_{17}H_{24}O_8NSe$	PM at *m/z* 450.0657	100	450.0662	-1.1	3.5	6.8
			$C_{11}H_{16}O_2NSe$	FI at *m/z* 274.0336	75	274.0341	-1.6		
			$C_{10}H_{14}NSe$	FI at *m/z* 228.0281	17	228.0286	-2.1		
			C_6H_7Se	FI at *m/z* 158.9707	7	158.9707	0.0		
			$C_6H_{12}ON$	FI at *m/z* 114.0916	42	114.0913	2.3		
**PySPent**	Parent compound	pHLS9, RU	$C_{13}H_{20}ONSe$	PM at *m/z* 286.0703	93	286.0705	-0.5	5.8	0.9
			$C_{10}H_{13}ONSe$	FI at *m/z* 243.0152	1	243.0157	-2.2		
			$C_9H_{11}OSe$	FI at *m/z* 214.9966	12	214.9970	-1.5		
			C_5H_3OSe	FI at *m/z* 158.9342	7	158.9343	-1.1		
			C_5H_5Se	FI at *m/z* 144.9549	22	144.9550	-1.2		
			$C_8H_{16}N$	FI at *m/z* 126.1278	100	126.1277	0.6		
			$C_4H_{10}N$	FI at *m/z* 72.0815	1	72.0808	9.9		
			C_4H_8N	FI at *m/z* 70.0659	4	70.0651	11		

M7	*N,N*-bis-Dealkylation	pHLS9, RU	$C_9H_{14}ONSe$	PM at *m/z* 232.0232	12	232.0234	-1.2	5.3	4.7
			$C_9H_{12}NSe$	FI at *m/z* 214.0126	82	214.0129	-1.5		
			C_6H_6NSe	FI at *m/z* 171.9657	25	171.9659	-1.6		
			C_5H_5Se	FI at *m/z* 144.9549	23	144.9550	-1.2		
			$C_4H_{10}N$	FI at *m/z* 72.0815	100	72.0807	11		
M8	*N,N*-bis-Dealkylation & *N*-oxygenation	RU	$C_9H_{14}O_2NSe$	PM at *m/z* 248.0184	10	248.0184	-0.2	6.7	0.9
			$C_9H_{12}ONSe$	FI at *m/z* 230.0077	10	230.0079	-0.7		
			C_5H_3OSe	FI at *m/z* 158.9343	12	158.9343	-0.4		
			$C_4H_{10}ON$	FI at *m/z* 88.0763	100	88.0757	6.8		
M23	Reduction	pHLS9	$C_{13}H_{22}ONSe$	PM at *m/z* 288.0870	25	288.0861	3.1	6.1	1.2
			$C_{13}H_{20}NSe$	FI at *m/z* 270.0749	100	270.0755	-2.4		
			$C_{10}H_{13}NSe$	FI at *m/z* 227.0202	49	227.0208	-2.4		
			$C_9H_{13}Se$	FI at *m/z* 201.0173	10	201.0177	-2.2		
			$C_8H_{16}N$	FI at *m/z* 126.1276	15	126.1277	-1.2		
			C_4H_8N	FI at *m/z* 70.0658	19	70.0651	9.7		
M24	Hydroxylation & oxidation	pHLS9, RU	$C_{13}H_{18}O_2NSe$	PM at *m/z* 300.0497	100	300.0497	0.1	5.7	4.6
			C_5H_5Se	FI at *m/z* 144.9549	4	144.9550	-1.1		
			$C_8H_{14}ON$	FI at *m/z* 140.1071	39	140.1070	0.6		
			C_5H_8ON	FI at *m/z* 98.0606	11	98.0600	6.0		
			C_4H_8ON	FI at *m/z* 86.0604	3	86.0600	3.8		
M25	Hydroxylation & oxidation	pHLS9	$C_{13}H_{18}O_2NSe$	PM at *m/z* 300.0484	17	300.0497	-4.5	9.2	0.8
			C_5H_5Se	FI at *m/z* 144.9549	3	144.9550	-1.6		
			$C_8H_{14}ON$	FI at *m/z* 140.1065	100	140.1070	-3.8		
			C_5H_8ON	FI at *m/z* 98.0601	28	98.0600	0.7		
			C_4H_8ON	FI at *m/z* 86.0603	6	86.0600	3.2		
M26	Hydroxylation	pHLS9	$C_{13}H_{20}O_2NSe$	PM at *m/z* 302.0651	56	302.0654	-1.0	4.9	1.1
			$C_{13}H_{18}ONSe$	FI at *m/z* 284.0545	32	284.0548	-1.1		
			C_5H_3OSe	FI at *m/z* 158.9343	100	158.9344	-0.6		
			$C_8H_{16}ON$	FI at *m/z* 142.1227	22	142.1226	0.2		
			C_4H_8ON	FI at *m/z* 86.0606	1	86.0600	6.5		
M27	Hydroxylation	pHLS9, RU	$C_{13}H_{20}O_2NSe$	PM at *m/z* 302.0650	28	302.0654	-1.3	5.1	2.0
				FI at *m/z* 230.9917	2	230.9919	-0.6		

			$C_9H_{11}O_2Se$	FI at *m/z* 160.9499	2	160.9500	-0.5		
			C_5H_5OSe	FI at *m/z* 126.1278	100	126.1277	0.6		
			$C_8H_{16}N$	FI at *m/z* 72.0815	9	72.0808	10		
			$C_4H_{10}N$						
M28	Hydroxylation	pHLS9, RU	$C_{13}H_{20}O_2NSe$	PM at *m/z* 302.0651	98	302.0654	-1.0	5.5	1.4
			$C_{13}H_{18}ONSe$	FI at *m/z* 284.0549	6	284.0548	0.3		
			$C_9H_{11}OSe$	FI at *m/z* 214.9966	13	214.9970	-1.5		
			C_5H_3OSe	FI at *m/z* 158.9342	13	158.9344	-1.2		
			$C_8H_{16}ON$	FI at *m/z* 142.1226	100	142.1226	-0.1		
			C_4H_8ON	FI at *m/z* 86.0606	2	86.0600	6.2		
M29	*N*-Oxygenation	pHLS9	$C_{13}H_{20}O_2NSe$	PM at *m/z* 302.0649	100	302.0654	-1.4	6.6	1.6
			$C_9H_{11}OSe$	FI at *m/z* 214.9962	2	214.9970	-3.3		
			C_5H_3OSe	FI at *m/z* 158.9343	29	158.9344	-0.2		
			$C_8H_{16}N$	FI at *m/z* 126.1278	89	126.1277	0.5		
			C_4H_8ON	FI at *m/z* 86.0606	85	86.0600	6.8		
M30	Reduction & hydroxylation	pHLS9	$C_{13}H_{22}O_2NSe$	PM at *m/z* 304.0805	100	304.0810	-1.9	5.7	2.6
			$C_{13}H_{20}ONSe$	FI at *m/z* 286.0701	25	286.0705	-1.3		
			$C_{13}H_{18}NSe$	FI at *m/z* 268.0594	4	268.0599	-2.0		
			$C_9H_{12}NSe$	FI at *m/z* 214.0126	22	214.0129	-1.4		
			C_5H_5Se	FI at *m/z* 144.9549	15	144.9550	-1.4		
			$C_8H_{18}ON$	FI at *m/z* 144.1382	34	144.1383	-0.7		
M31	Dihydroxylation	RU	$C_{13}H_{20}O_3NSe$	PM at *m/z* 318.0604	36	318.0603	0.4	4.7	5.1
			$C_9H_{11}O_2Se$	FI at *m/z* 230.9918	2	230.9919	-0.5		
			$C_5H_3O_2Se$	FI at *m/z* 174.9292	2	174.9293	-0.6		
			$C_8H_{16}ON$	FI at *m/z* 142.1228	100	142.1228	1.1		
			$C_4H_{10}ON$	FI at *m/z* 88.0763	13	88.0763	7.5		
			C_4H_8N	FI at *m/z* 70.0660	2	70.0660	12		
M32	Dihydroxylation	pHLS9, RU	$C_{13}H_{20}O_3NSe$	PM at *m/z* 318.0606	100	318.0603	1.1	5.9	4.8
			$C_{13}H_{18}O_2NSe$	FI at *m/z* 300.0500	31	300.0497	0.8		
			$C_{13}H_{16}ONSe$	FI at *m/z* 282.0394	9	282.0392	0.8		
			C_9H_9Se	FI at *m/z* 196.9866	9	196.9864	1.2		
			$C_8H_{16}O_2N$	FI at *m/z* 158.1176	32	158.1176	0.4		

			$C_8H_{14}ON$	FI at *m/z* 140.1071	62	140.1070	0.6		
			C_5H_8ON	FI at *m/z* 98.0606	27	98.0600	5.7		
			$C_4H_7O_2$	FI at *m/z* 87.0448	31	87.0441	8.2		
M33	Hydroxylation & glucuronidation	RU	$C_{19}H_{28}O_8NSe$	PM at *m/z* 478.0975	11	478.0975	0.1	3.6	6.7
			$C_{13}H_{20}O_2NSe$	FI at *m/z* 302.0652	56	302.0654	-0.7		
			$C_9H_{11}O_2Se$	FI at *m/z* 230.9924	1	230.9919	-0.1		
			C_5H_5OSe	FI at *m/z* 160.9499	1	160.9500	-0.5		
			$C_8H_{16}N$	FI at *m/z* 126.1279	100	126.1277	1.4		
			$C_4H_{10}N$	FI at *m/z* 72.0816	2	72.0808	12		
M34	Hydroxylation & glucuronidation	pHLS9, RU	$C_{19}H_{28}O_8NSe$	PM at *m/z* 478.0975	100	478.0975	0.1	5.3	6.4
			$C_{13}H_{20}O_2NSe$	FI at *m/z* 302.0656	67	302.0654	0.7		
			$C_{13}H_{18}ONSe$	FI at *m/z* 284.0545	3	284.0548	-1.0		
			$C_9H_{11}OSe$	FI at *m/z* 214.9970	11	214.9970	0.2		
			C_5H_3OSe	FI at *m/z* 158.9344	8	158.9344	0.4		
			$C_8H_{16}ON$	FI at *m/z* 142.1228	62	142.1226	1.2		
			C_4H_8ON	FI at *m/z* 86.0608	1	86.0600	8.3		

Table S3. General involvement of monooxygenases in the formation of the given ASProp, MASProp, MASPent, PySProp, and PySPent single-step phase I metabolites. Pooled human liver microsomes (pHLM) incubations were used as positive control. Metabolite IDs correspond to Table S1. CYP, cytochrome P450; FMO, flavin-containing monooxygenase, +, detected in in vitro incubations; -, not detected in in vitro incubations.

Parent compound	CYP										FMO	pHLM
Metabolite ID	1A2	2A6	2B6	2C8	2C9	2C19	2D6	2E1	3A4	3A5	3	
ASProp												
M1 (dihydro-)	-	-	-	-	-	-	-	-	-	-	-	+
MASProp												
M3 (*N*-demethyl-)	-	+	+	-	-	-	+	+	-	-	-	+
M4 (dihydro-)	-	-	-	-	-	-	-	-	-	-	-	+
M5 (hydroxy-)	-	-	+	-	-	+	+	-	-	-	-	+
M6 (hydroxylamine)	-	+	+	-	-	+	-	-	+	-	-	+
MASPent												
M7 (*N*-demethyl-)	-	+	+	-	-	-	+	-	+	-	-	+
M9 (dihydro-)	-	-	-	-	-	-	-	-	-	-	-	+
M10 (hydroxy-)	-	-	+	-	-	+	+	-	-	-	-	+
M11 (hydroxy-)	-	-	-	-	-	+	+	-	-	-	-	+
M12 (hydroxylamine)	-	+	-	-	-	-	-	-	+	+	-	+
PySProp												
M3 (*N,N*-bis-dealkyl-)	-	+	+	-	-	+	+	-	+	+	-	+
M13 (dihydro-)	-	-	-	-	-	-	-	-	-	-	-	+
M14 (oxo-)	-	-	-	-	-	-	-	-	-	-	-	-
M15 (oxo-)	-	+	+	-	-	-	-	-	+	-	-	+
M16 (hydroxy-)	-	+	+	-	-	+	-	-	+	+	-	+
M17 (hydroxy-)	-	-	+	-	-	+	+	-	-	-	-	+
M18 (hydroxy-)	-	-	+	-	-	-	-	-	-	-	-	+
M19 (hydroxy-)	-	+	+	-	-	+	-	-	+	+	-	+
M20 (*N*-oxide)	-	-	-	-	-	-	-	-	+	-	+	+
PySPent												
M7 (*N,N*-bis-dealkyl-)	-	-	+	-	-	+	+	-	+	+	-	+

M23 (dihydro-)	-	-	-	-	-	-	-	-	-	-	-	+
M24 (oxo-)	-	-	-	-	-	-	-	-	+	-	-	+
M25 (oxo-)	+	-	+	-	-	+	+	-	+	+	-	+
M26 (hydroxy-)	+	-	+	+	-	-	+	-	+	+	-	+
M27 (hydroxy -)	+	-	+	-	+	+	+	-	-	-	-	+
M28 (hydroxy-)	-	+	+	+	-	-	-	-	-	-	-	+
M29 (*N*-oxide)	-	-	-	-	-	-	-	-	+	+	+	+
